# ZIP1-dependent lymphatic adaptations facilitate lymph node metastasis of pancreatic cancer

**DOI:** 10.7150/ijbs.134874

**Published:** 2026-09-10

**Authors:** Ruoyang Chen, Kaili Zhang, Jiajia Wan, Yuanyuan Yang, Linlin Wang, Xiangjing Miao, Qingqing Fang, Yu Hu, Xixi Duan, Xiaohan Lou, Xiaohan Yao, Anqi Li, Linyu Zhu, Zhihai Qin, Chen Ni

**Affiliations:** Medical Research Center, Henan China-Germany International Joint Laboratory of Tumor Immune Microenvironment and Disease, the First Affiliated Hospital of Zhengzhou University, Zhengzhou, Henan 450052, China.; R. Chen, K. Zhang, J. Wan and C. Ni contributed equally to this article.

**Keywords:** pancreatic cancer, lymph node metastasis, lymphatic endothelial cell, glycolysis, Claudin-5, ZIP1

## Abstract

Tumor disseminates through lymphatic vessels to form lymph node (LN) metastasis, which requires lymphatic adaptations. However, it remains largely unknown how lymphatic vessels adapt to the stimuli causing LN metastasis of pancreatic cancer. Zinc dyshomeostasis has been observed in various tumors, including pancreatic cancer. We hypothesize that lymphatic vessels actively adapt to zinc imbalance, and examine lymphatic functions and LN metastasis of transplanted pancreatic cancer in zinc transporter ZIP1 deficient mice. The results show that ZIP1 is required for lymphatic glycolysis and junctional adaptations that facilitate LN metastasis of pancreatic cancer. In *Zip1^-/-^* mice, the opening of lymphatic barrier and LN metastasis of pancreatic cancer are both mitigated. Inhibition of ZIP1 decreases Zn^2+^ influx and increases tight junction protein Claudin-5 in lymphatic endothelial cells, thereby suppressing tumor trans-endothelial migration. Mechanistically, ZIP1 promotes lymphatic glycolysis to produce lactic acids which impair Claudin-5 expression. Furthermore, in lymphatic-specific *Zip1* knockout mice, lymphatic barrier is tightened and LN metastases are reduced. Importantly, ZIP1^+^ lymphatics are positively associated with LN metastasis of human pancreatic cancer. Taken together, these findings suggest that ZIP1-dependent metabolic and junctional adaptations in lymphatics accelerate LN metastasis of pancreatic cancer, providing potential therapeutic targets for pancreatic cancer metastasis.

## Introduction

Pancreatic ductal adenocarcinoma (PDAC) is a highly aggressive and lethal malignancy, with a 5-year overall survival of 10% 1. Approximately 80-85% of patients present with either unresectable or metastatic disease at diagnosis 2. Even for patients diagnosed with a localized tumor, most patients relapsed after surgery with distant or lymph node (LN) metastasis ultimately 3, 4. Lymphatic metastasis constitutes a critical route for metastasis of pancreatic cancer, with tumor cells disseminating via diverse lymphatic routes to form LN metastasis in early stages 5, 6. The incidence of LN metastasis is strongly correlated with poor prognosis of PDAC 7, 8. Although the exact contribution of lymphatic metastasis of PDAC to distant metastasis is unclear, the entry of tumor cells in LN may serve as a seed for sequential distant metastasis, or suppress systemic immunity to promote distant metastasis 9-13. Originally, lymphatic vasculature is considered as a passive conduit for tumor transport, but experimental and clinicopathological studies suggest that lymphatic metastasis requires dynamic adaptation of lymphatic vessels, including lymphangiogenesis and lymphatic remodeling 9. In mouse model, Ang-2 promotes lymphangiogenesis and drives lymphatic metastasis of pancreatic cancer 14. In PDAC, lymphatic vessel density (LVD), especial peritumoral LVD, has been reported to be correlated with lymph node metastasis and survival of patients 8. However, a mechanistic understanding for the lymphatic adaptations that are required for lymph node metastasis of pancreatic cancer is still lacking.

The lymphatic vasculature is composed of an intricate network of lymphatic vessels that start in the tissue periphery as initial lymphatics that merge into bigger pre-collecting and collecting vessels 9. Lymphatic endothelial cells (LECs) forming initial lymphatics are loosely interconnected by button-like junctions that involve adherent junction proteins (e.g. VE-Cadherin) and tight junction proteins (e.g. Claudin-5) 15. Endothelial integrity defects may initiate lymphatic metastasis of cancer cells 16. Compared to normal LECs, LECs from high metastatic tumor alter gene expression of cell adhesion and junctional proteins 17. Disruption of endothelial lymphatic barrier by tumor-derived factors (e.g. VEGFC, VEGFD, PDGF-BB) increases permeability and facilitates the lymphatic metastatic spread of tumors 8, 18. A disruptive effect on lymphatic endothelium continuity by low-molecular-weight hyaluronan (LMW-HA) leads to a promotion on melanoma lymphatic metastasis 16. Recent studies suggest that the activation of metabolic pathways is critical for LEC development, maintenance, and function 19. Fatty acid β-oxidation in LECs is used for proliferation and for epigenetic regulation of lymphatic marker gene expression during LEC differentiation 19-21. Nevertheless, the metabolic controlling of lymphatic junction during LN metastasis is unknown.

Zinc is an essential trace element and catalytic/structural component used by many metalloenzymes and transcription factors, playing a role in the correct functioning of lipid and glucose metabolism 22. Physiologically, cellular zinc homeostasis is tightly regulated by zinc transporters including ZIP (SLC39A1-14) and ZnT (SLC30A1-10) family proteins 23. Zinc transporter ZIP1 (Zrt- and Irt-like protein 1, encoded by *SLC39A1*) mediates the influx of Zn^2+^ into the cell cytosol from the extracellular space 23-25. ZIP1 is abundantly expressed in a wide range of tissues and has been reported to be expressed by endothelial cells 25-27. In tumors, however, zinc disturbance is frequently observed, hypothetically attributing to high proliferating activity of tumor cells that requires excess zinc for use 23, 28. Our recent study showed that the expression of ZIP1 on fibroblasts regulates gap junctional formation and Zn^2+^ delivery to cancer cells 29. In the development of pancreatic cancer, zinc loss in epithelial cells occurs as a common and early event 28, 30. And studies reveal that ZIP4 is markedly upregulated in cancerous tissues, which enhances zinc accumulation and promotes the proliferation and metastasis of pancreatic cancer 28, 31, 32. But, how zinc dyshomeostasis contributes to LN metastasis of pancreatic cancer is unknown. A study reported that ZIP10 expressed in tumor cells was found to be significantly associated with the metastasis of breast cancer to the LN 33. Zinc deficiency has been linked to reduced lymphatic absorption of vitamin A and α-tocopherol 34, 35, implying that zinc is involved in regulation of lymphatic functions. Therefore, we postulated that lymphatic vessels would make dynamic adaptations to zinc imbalance in pancreatic cancer and facilitate LN metastasis.

Herein, we find that ZIP1-dependent metabolic and junctional adaptations promote LN metastasis of pancreatic cancer. Hypothesizing that LN metastasis required lymphatic adaptations to zinc dyshomeostasis, we established a popliteal LN metastasis model of pancreatic cancer in *Zip1^-/-^* mice, and found that LN metastasis was decreased in *Zip1^-/-^* mice when compared to *Zip1^+/+^* mice. As expected, the lymphatic intercellular barrier function was improved in *Zip1^-/-^* mice. Furthermore, ZIP1 in LECs promoted tumor trans-endothelial migration in Claudin-5-dependent manner *in vitro*. Mechanistic studies revealed that ZIP1 maintained high glycolysis in LECs to inhibit the expression of Claudin-5. Moreover, we established a popliteal LN metastasis model of pancreatic cancer in mice with lymphatic-specific knockout of *Zip1* (*Zip1^flox/flox^*; *Lyve1-Cre+, Zip1^flox/+^*; *Prox1-Cre+, Zip1^flox/flox^*; *Prox1-Cre+*), validating that lymphatic ZIP1 deficiency enhanced lymphatic barrier function and inhibited LN metastasis. Finally, multiplex immunostaining of human PDAC tumor tissues suggested the association of ZIP1-expressing lymphatics with LN metastasis. Taken together, our results demonstrate a mechanism for ZIP1-mediated adaptation of lymphatic vessels that enhances lymph node metastasis of pancreatic cancer, and provide potential therapeutic targets for clinical PDAC treatment.

## Materials and Methods

### Study approval

All experiments were performed in accordance with local guidelines and regulations. This study was reviewed and approved by the Ethics Committee of Scientific Research and Clinical Trial at the First Affiliated Hospital of Zhengzhou University (2021-KY-0626-002). The application of TMA (tumor tissue microarray) of cancer patients was approved by the Ethics Committee of Outdo Biotech (SHYJS-BC-2310001). The clinical tumor staging in this study was based on the 8th edition of the AJCC (American Joint Committee on Cancer) Cancer Staging Manual.

### Animals

*Zip1^-/-^* mice on C57BL/6 background were purchased from Cyagen (KOCMP-30791-Slc39a1-B6N; Suzhou, China), as described in the previous report 29. Mice with floxed *Zip1* (*Zip1^flox/flox^*) on C57BL/6 background were purchased from Cyagen (CKOCMP-30791-Slc39a1-B6J; Suzhou, China) to produce LEC-specific conditional *Zip1* knockout (*Zip1^ΔLYVE1^, Zip1^flox/+^*; *Prox1-Cre+, Zip1^flox/flox^*; *Prox1-Cre+*) mice. The tissue-specific gene deletion of *Zip1* was confirmed on mRNA level by the following primers: F1: 5'-TAGGGTCACTTACATGAACACGA-3'; R1: 5'-CACATCCCACTTTCTTGAGAACATC-3'. Mice expressing *Cre*-recombinase under the control of the *Lyve1* promoter (*Lyve1-Cre*) and *Prox1* promoter (*Prox1-Cre*) on C57BL/6 background were developed by Cyagen Biosciences (Santa Clara, CA, USA) 36. For mosaic deletion of *Zip1* in LECs, *Zip1^flox/flox^* mice were crossed with *Lyve1-Cre* or *Prox1-Cre* mice to obtain LEC-specific *Zip1^ΔLYVE1^, Zip1^flox/+^*; *Prox1-Cre+, Zip1^flox/flox^*; *Prox1-Cre+* mice. All mice were housed at 21°C and 50% humidity on average, on a 12 h light/dark cycle in a specific pathogen-free facility at Zhengzhou University. Our study examined male mice because male animals exhibited less variability in phenotype.

### Cell lines

The SVEC4-10 cell line was provided in kind by Professor Mingzhao Zhu from the Chinese Academy of Sciences, as previously described 36. Human lymphatic endothelial cells (hLEC) were acquired from iCell Bioscience and cultured in a primary endothelial cell culture system (PriMed-icell-002), which consisted of ECM, 5% fetal bovine serum, and a supplement containing 10 ng/ ml Rh VEGF, 5 ng/ml Rh EGF, 5 ng/ml Rh FGF, 15 ng/ ml Rh IGF-1, as previously described 36. KPC-Luc cell lines were generated from spontaneous cancer of *LSL-Kras^G12D/+^;LSL-Trp53^R172H/+^;Pdx-1-Cre* (KPC) mice and kindly provided by Dr Xiaomeng Liu from Fudan University, as previously described 37. Mouse pancreatic cancer Pan02 and KPC-Luc, human pancreatic cancer PANC-1 were grown in DMEM supplemented with 10% FBS, and 100 IU/ml penicillin-streptomycin. Cells were incubated at 37°C in a humidified atmosphere of 5% CO_2_ and 95% air. The stable Pan02-GFP-Luc cell line was constructed by transfecting Pan02 cells with a GFP-Luc lentivirus vector (Genechem, Shanghai, China). S100A4-KO Pan02-GFP-Luc cells were generated using CRISP/cas9 system as previously reported 36.

### Popliteal LN metastasis study

A mouse model of popliteal LN metastasis of pancreatic cancer was generated as reported previously 38. Briefly, 1 × 10^6^ GFP-Luc-labeled Pan02 or KPC-luc cells were inoculated into the footpad of 6-8-week-old male mice (5-6 mice in each group, repeated at least three times). The tumor volume was measured every 2 days. Tumor volumes (V) were assessed in mm^3^ using the formula: V = 0.5 × (L × W^2^), with L being the long and W being the short diameter of the tumor. Mice were sacrificed at indicated time. The primary tumor in the footpad was collected for immunohistochemical analysis. Popliteal LN and lung were removed for bioluminescence quantification using the IVIS Spectrum Imaging System (PerkinElmer). The region of interest (ROI) of the organs was evaluated with Living Image 4.4. Popliteal LNs were fixed with 4% paraformaldehyde compound for the paraffin section procedure and staining with hematoxylin-eosin (HE).

### Lymphatic draining into LN

1 μL FITC-Dextran (2 mg/mL, 70 kD) was injected into the right ear while the left ear was injected with PBS as a control. After 30 min, the cervical LNs were harvested from the sacrificed mice and the fluorescence in LNs was then examined using the IVIS Spectrum Imaging System (PerkinElmer).

### Immunofluorescence staining and immunohistochemistry

Frozen sections (6 μm) from isolated tissues were prepared for immunofluorescence (IF) staining, and stained for LYVE1 (1:200, MAB2125-100, R&D), PROX1 (1:200, AF2727, R&D), Claudin-5 (1:200, 34-1600, Invitrogen), VE-Cadherin (1:200, AF1002, R&D) and ZO-1 (1:200, 40-2200, Invitrogen). The images were acquired with the Vectra machine (Perkin Elmer, Waltham, MA, USA). For quantitative analysis of the lymphatic vessels, 20-30 optical fields were taken for each section (200 × magnification), with 3 tumors selected randomly in each group. The positive area was quantified with ImageJ (Media Cybernetics, Bethesda, MD, USA). Immunohistochemistry (IHC) staining of paraffin-embedded sections was conducted as described previously 39.

Multiplex immunofluorescence staining of tumor tissue microarray (TMA) was performed as previously described 29. A human PDAC TMA with survival data was purchased from Outdo Biotech (HPanA170Su05, Shanghai, China). The TMA represented 88 PDAC cases. Briefly, tissue sections were deparaffinized and incubated in Tris-EDTA at 95 °C for 20 min for antigen retrieval. Co-staining of LYVE1 (1:20,000, Ab219556, abcam), ZIP1 (1:100, AZT-001, Alomone, Israel) and Claudin-5 (1:200, 34-1600, Invitrogen) in TMAs was performed according to the manufacturer's instructions using a PANO multiplex IHC 6-colour kit (PN100718AD, PANOVUE, Beijing, China). Tissue sections were incubated overnight at 4°C with primary antibody for LYVE1, 3 h at room temperature for ZIP1 and 2 h at room temperature for Claudin-5. LYVE1 was labelled with PPD620, ZIP1 with PPD650, and Claudin-5 with PPD520. The results of immunofluorescence staining were acquired and assessed with a Vectra (Perkin Elmer). The percentage of immunoreactive cells and staining intensity were evaluated using the Inform software. LEC was identified according to LYVE1 staining. The staining area of LYVE1^+^ LEC, or the staining area of ZIP1^+^LYVE1^+^ LEC in each specimen was defined as low or high.

### Trans-endothelial migration assay

Confluent SVEC4-10, mouse primary LEC, SVEC4-10^ctrl^ and SVEC4-10^kd^ layers grown in flasks were gently detached with trypsin, and 1 × 10^5^ cells (SVEC4-10, SVEC4-10^ctrl^ and SVEC4-10^kd^) or 2 × 10^5^ primary cells (mLECs) were seeded on the upper surface of a transwell insert (NEST Insert, 724301). After one day of culture, LECs formed a complete cell monolayer. For cell migration, 3 × 10^5^ Pan02 cells stained with fluorescent dyes (CMFDA, 40721ES60, YEASEN) were added in a volume of 500 μL to the upper chamber of a 12-well transwell plate with an 8 μm pore (for carcinoma cells) in the inserts (NEST Insert, 724301). The lower chamber contains culture medium with high concentration of serum (20%). The number of cells that migrated to the lower well after 16 h or 24 h was counted with confocal microscopy 40.

### Seahorse metabolic measurements

To evaluate the extracellular acidification rate (ECAR), the Seahorse XF96 Extracellular Flux Analyzer (Seahorse Bioscience, MA, USA) was used. SVEC4-10 (6,000 cells/well) were seeded onto the cell culture plate and incubated overnight. On the next day, the cells were washed twice with XF base medium containing 200 mM L-glutamine. The plate was then put into a non-CO_2_ incubator at 37°C and incubated for 1 h. To measure ECAR, 10 mM glucose, 1 µM oligomycin and 50 mM 2-deoxyglucose (2-DG) were sequentially auto-injected into each well, followed by 3 measurement cycles. ECARs were normalized according to Hoechst (Beyotime) staining. The assays were carried out with 5-6 replicates for each group, and each experiment was repeated independently at least three times 36. For measurement of mitochondrial respiration, the blockers were added sequentially through the ports of the Seahorse Flux Pak cartridges. Oxygen consumption rates were measured using a Seahorse XF96 extracellular flux analyzer 41.

### Assessment of paracellular tightness

Paracellular tightness of the LEC monolayer was determined by measuring the electrical impedance of LEC monolayer using an xCELLigence System RTCA-MP instrument (ACEA Biosciences Inc., San Diego, CA) 42. Briefly, 40,000 cells were seeded into the 96-well E-Plate (ACEA Biosciences Inc.) and monitored using the RTCA-MP instrument. Each group included at least three replicates. Once cell index values reached their peak values, indicating that LECs have formed a monolayer, stimulators were added to the culture. The cell index value on stimulator application was adopted as a reference point. The impedance was analyzed as normalized cell index and presented as relative cellular impedance. All experiments were repeated at least three times.

### Permeability assay

1 × 10^5^ cells (SVEC4-10^ctrl^ and SVEC4-10^kd^) or 2 × 10^5^ primary cells (mLECs) were seeded on the upper surface of a transwell insert. After one day of culture, LECs formed a complete monolayer. 100 μL DMEM containing 70 kD FITC-Dextran (2 mg/mL, Sigma-Aldrich, Bornem, Belgium) was added to the upper chamber of the transwell and 500 μL DMEM to the lower chamber. After 30 min at 37℃ in the incubator, the medium in the lower chamber was mixed and 100 μL of it was added into 96-well plates (3 replicates for each sample), and the fluorescence (Ex: 488 nm, Em: 530 nm) was measured by SpectraMAX i3X (Molecular Devices, CA, USA).

### Western-blotting analysis

Cells were lysed with RIPA (Solarbio, China) lysis buffer and the lysates were collected. Proteins were separated by 10% SDS-PAGE and transferred onto Nitrocellulose (NC) membranes. The membrane was incubated with the primary antibodies, and detected with HRP-conjugated secondary antibody (ABclonal). The following primary antibodies were used: ZIP1 (1:1000, AL0-AZT-001; Alomone, Israel), Claudin-5 (1:1000, 34-1600, Invitrogen, USA), VE-Cadherin (1:1000, ab33168, Abcam, UK), ZO-1 (1:1000, 40-2200, Invitrogen, USA), GLUT-1 (1:1000, 12939S, CST, USA), HK1 (1:1000, ab154839, Abcam, UK), HK2 (1:1000, 2867S, CST, USA), p-PFKFB2 (1:1000, 13064S, CST, USA), PFKFB2 (1:1000, TA314335, OriGene, USA), β-actin (1:1000, AC026, ABclonal, CN), p-mTOR (1:1000, 2971S, CST, USA), mTOR (1:1000, 2972S, CST, USA), p-S6K (1:1000, F2333, Selleck, USA), S6K (1:1000, F3527, Selleck, USA), p-Akt (1:1000, 4060S, CST, USA), Akt (1:1000, 2920S, CST, USA), p-p65 (1:1000, 3033S, CST, USA), p65 (1:1000, 8242S, CST, USA), p-ERK (1:1000, 4377S, CST, USA), ERK (1:1000, 5013S, CST, USA), p-p38 (1:1000, 4511S, CST, USA), p38 (1:1000, 8690S, CST, USA).

### Flow cytometry

Popliteal LNs were removed from Pan02-GFP-Luc tumor-bearing mice and cut into small pieces for digestion. GFP^+^ tumor cells were examined by flow cytometry (Canto II with BD FACSDiva software v8.0.1), and the results were analyzed using FlowJo v10 (BD Bioscience, USA).

### Isolation of primary mouse LECs

Primary mouse LECs were isolated from mouse LNs. Firstly, LNs were separated and digested in DMEM containing 1 mg/mL collagenase IV (Sigma, C5138) and 40 μg/ml DNase I (Worthington, LS002139) for 30 min at 37°C. In the second step, the upper layer of the liquid mixture that has been digested in the first step is suctioned off, and the remaining components continue to be digested in DMEM containing 1 mg/mL collagenase D (Roche,11088866001) and 40 μg/ml DNase I for 30 min at 37°C, with multiple blowing and mixing in the process. Finally, the digestion is terminated, followed by centrifugation, and cells were then seeded onto collagen-coated dishes. After 24 h, non-adherent cells were removed, and adherent cells were cultured until cell monolayers were 80-90% confluent. The rest of the isolation methods were referred to previous literature report 43. Primary mouse lymphatic endothelial cells were cultured in a primary endothelial cell culture system (PriMed-icell-002). Isolated primary lymphatic endothelial cells identified by immunostaining for the LEC markers LYVE1 (1:200, MAB2125-100, R&D) and PROX1 (1:200, AF2727, R&D).

### CRISPR/Cas9 assay

*Zip1* knockdown SVEC4-10^kd^ cell line was generated using single guide CRISPR-Cas9 (Cas9/sgRNA)-mediated deletions. Two sgRNAs targeting sequences were selected as follows: ZIP1-sgRNA-1-F, 5'-ACAGCCACCATGGGGCCCTG-3'; ZIP1-sgRNA-1-R, 5'-CAGGGCCCCATGGTGGCTGT-3'; ZIP1-sgRNA-2-F, 5'- CTGGAGTTCCGCCGGCCTGG-3'; and ZIP1-sgRNA- 2-R, 5'- CCAGGCCGGCGGAACTCCAG-3'. The sgRNA cloning vector is pSpCas9-2A-Puro (PX459). PX459 plasmid ligated with/without sgRNAs and packaging plasmids were co-transfected into 293T cells. The virus was collected after 72 h and was used to infect SVEC4-10 cells for 36 h. Cell limited dilution were seeded into 96-well plates to obtain single cell clones. Monoclonal cells were selected and screened using the western immunoblot technique. Successful *Zip1* knockdown in SVEC4-10 cells (SVEC4-10^kd^) were used for the following experiments.

### RNA interference

Small interfering RNA (siRNA) oligonucleotides targeting mouse *claudin-5* were synthesized by Sangon Biotech (Shanghai, China). Small interfering RNA (siRNA) oligonucleotides targeting mouse *Zip1* were purchased from Santa Cruz Biotechnology. Small interfering RNA (siRNA) oligonucleotides targeting human *ZIP1* were purchased from GenePharma (Shanghai, China): si-1, 'GGCUUACAAGGAGCAGUCATT'; si-2, 'CUGAGCCUAGUAAGCUGUUTT'; si-3, 'CUGGCACCUUUCUCUAUAUTT'; si-4, 'GCCUGCUCUUCAUCCAAAUTT'. SVEC4-10 cells, SVEC4-10^kd^, or hLEC cells were transfected with siRNA oligonucleotides using jetPRIME® (101000046, Polyplus, Shanghai, China) according to the manufacturer's protocol.

### RNA isolation and quantitative RT-PCR

Total RNA was extracted using FastPure Cell/Tissue Total RNA Isolation Kit V2 (RC112-01, Vazyme), and cDNA was synthesized using a reverse transcription kit (RR036A, Takara), following manufacturer's instructions. Quantitative real time PCR was performed using TB Green® Premix Ex Taq™ II (Tli RNase H Plus) (RR820L, Takara) in a Bio-Rad CFX384 Real-Time 384-well PCR qPCR Detection System. Primers used in this study are listed in **Table S3**. mRNA expression was calculated using the 2^-ΔΔCt^ method, and 18S rRNA was used as a reference for gene expression. Each assay was independently repeated at least three times.

### Zn^2+^ uptake

To detect Zn^2+^ uptake by cells, 1 × 10^4^ SVEC4-10^ctrl^ or SVEC4-10^kd^ were seeded into 96-well black plates and cultured overnight. The cells were loaded with 2 µM FluoZin3 for 1 h, and then 30 µM ZnCl_2_ was added in HBSS. Sixty seconds after addition, FluoZin3 fluorescence was read every 30 s to evaluate intracellular labile Zn^2+^ in cells. All values minus the values of the blank group were used to estimate intracellular Zn^2+^. Time point of cell fluorescence (F) at the beginning were used as F0, and the alteration in intracellular Zn^2+^ was calculated as ΔF/F0, where ΔF = F-F0. The origin was forced to zero, and 60 s was applied to the start time of the measurement 29.

### Glucose analog uptake

2-[N-(7-Nitrobenz-2-oxa-1,3-dioxol-4-yl)amino]-2-deoxyglucose (2-NBDG) is a glucose analog. SVEC4-10^ctrl^/SVEC4-10^kd^ cells were seeded in a 24 well plate. After overnight culture, the culture medium was discarded and cells were washed with PBS (pH 7.4). This was followed by the addition of low-glucose culture media supplemented with 2-NBDG (100 μM, N13195, Life Technologies) and an incubation of 45 min at 37°C. Next, cells were washed with PBS and trypsin was added, and they were harvested, centrifuged at 1,500 rpm for 5 min at 4 °C, washed twice with ice-cold PBS, and kept on ice. A control sample lacking 2-NBDG was used to set the blank in the flow cytometer and gate parameters for 2-NBDG detection.

### RNA sequencing analysis

Total RNA from SVEC4-10^ctrl^ and SVEC4-10^kd^ cells was isolated using TRIzol reagent and subjected to bulk RNA-sequencing (RNAseq). RNAseq analysis was performed on an Illumina NovaSeq6000 by Gene Denovo Biotechnology Co. (Guangzhou, China). Three biological replicates were performed for each group of cells. Differential expression analysis between two different groups was performed with DESeq2 software. Genes with a false discovery rate (FDR) below 0.05 and |log2FC|>1 were considered differentially expressed genes (DEGs), and GO and KEGG enrichment of the identified DEGs was conducted. The raw sequence data reported in this paper have been deposited in the Genome Sequence Archive of the National Genomics Data Center (NGDC), China National Center for Bioinformation/Beijing Institute of Genomics, Chinese Academy of Sciences, under accession number CRA015207.

### Statistical analysis

Statistical analyses were performed using GraphPad Prism (GraphPad Software, La Jolla, CA, USA). The unpaired Student's t-test (two-tailed) was used to evaluate statistical significance between two groups. Multiple groups were compared by one-way ANOVA, and curves comparisons were using two-way ANOVA. Pathological parameter analysis of human patients was performed using SPSS Statistics (IBM, NY, USA). Survival curves were examined using the log-rank test. Contingency data were analysed using Fisher's exact tests. Correlations were estimated using Pearson's correlation analysis. The data are presented as mean ± standard error of the mean (SEM). Sample sizes are indicated where appropriate. Asterisks (*) denote statistical significance (*p < 0.05; **p < 0.01; ***p < 0.001).

## Results

### Host ZIP1 is required for LN metastasis of pancreatic cancer

Considering the common existence of metastasis-to-metastasis (including LN metastasis) in pancreatic cancer^44^, and the feasibility to quantifying LN metastasis, the popliteal LN metastasis model in which cancer cells are injected into mouse footpads and allowed to metastasize to LN, was adopted in our study. To study the effect of host ZIP1 on LN metastasis of pancreatic cancer, we constructed a popliteal LN metastasis model of pancreatic cancer by injecting Pan02 tumor cells labeled with green fluorescent protein (GFP) and luciferase (Luc) (Pan02-GFP-Luc) into the footpads of wild-type littermates (*Zip1^+/+^*) and *Zip1^-/-^* mice (**Figure 1, A and B**). Tumor growth was monitored and tumor metastasis to LNs was examined after 35 days (**Figure 1B**). Using bioluminescence imaging, a dramatic decrease of LN metastasis to the ipsilateral popliteal LNs, was observed in *Zip1*^-/-^ mice (5/18, 27.8%) when compared to *Zip1^+/+^* mice (12/18, 66.7%) (**Figure 1C-E**), while tumor growth of primary tumor was similar between the two groups (**Figure 1F**). No metastasis was observed in the contralateral popliteal LNs from both mouse strains (**Figure 1G**). Noteworthily, lung metastasis was also significantly decreased in *Zip1*^-/-^ mice when compared to *Zip1^+/+^* mice (**Figure 1, H and I**). Furthermore, we performed Hematoxylin-eosin (HE) staining and confirmed the LN metastasis of tumor cells (**Figure 1K**). Benefiting by the expression of GFP, we were able to detect LN metastasis by flow cytometry. The results showed that a large number of tumor cells were observed in draining LNs of *Zip1^+/+^* mice, while rare tumor cells were found in that of *Zip1*^-/-^ mice (**Figure 1, L and M**). These results suggested that host ZIP1 deficiency reduces tumor metastasis of pancreatic cancer to the sentinel LNs.

In an alternative pancreatic cancer model, we transplanted KPC-Luc tumor cells into the footpads of *Zip1^+/+^* and *Zip1*^-/-^ mice (**Figure S1, A and B**). KPC-Luc cells were established from spontaneous pancreatic cancer of *LSL-Kras^G12D/+^;LSL-Trp53^R172H/+^;Pdx-1-Cre* (KPC) mice. Consistent with above observations, tumor metastasis in ipsilateral popliteal LNs of *Zip1*^-/-^ mice was largely reduced compared to that of *Zip1^+/+^* mice, while tumor growth of KPC-Luc tumor was similar between *Zip1^+/+^* and *Zip1*^-/-^ mice (**Figure S1, C**-**E**). Different from Pan02-GFP-Luc cells, lung metastasis of KPC-Luc cells was not frequent in both *Zip1^+/+^* and *Zip1*^-/-^ mice (**Figure S1, F and G**). HE staining confirmed the LN metastasis of KPC-Luc tumor cells (**Figure S1, H**).

### ZIP1 controls the proper opening of lymphatic barrier

Lymphatic vessels constitute an important route for LN metastasis. Lymphatic remodeling, including lymphangiogenesis and structural changes of the initial and collecting lymphatic vessels, occurs as a response to cues that drive tumor cells to invade lymphatic vessels. We first examined lymphatic vessel density and junctional protein expression on lymphatic vessels in pancreatic cancer. The results showed that LYVE1^+^ lymphatic vessels mainly distribute around the tumor of Pan02-GFP-Luc and the lymphatic vessel density (LVD) was not significantly different between tumors from *Zip1^+/+^* and *Zip1*^-/-^ mice (**Figure 2, A and B**). Another LEC marker, PROX1 was co-expressed in LYVE1^+^ LECs, and also distributed similar in tumors from *Zip1^+/+^* and *Zip1*^-/-^ mice (**Figure 2C**). Interestingly, the expression of the tight junction protein Claudin-5 on the lymphatic vessels was upregulated in tumors from *Zip1*^-/-^ mice compared to that from *Zip1^+/+^* mice, while the expression of ZO-1 and VE-Cadherin were not affected (**Figure S2, A and B**). We further examined the lymph draining in each group by injecting 70 kD FITC-Dextran at the ear and detecting the fluorescence in the cervical lymph node. The results showed that FITC-Dextran was efficiently drained into the cervical lymph nodes of *Zip1^+/+^* mice, and the draining was largely blocked in that of *Zip1*^-/-^ mice (**Figure 2, F and G**). These results suggest ZIP1 controls the proper opening of lymphatic barrier.

On what type of lymphatics, initial or collecting lymphatics, intercellular junctions are affected by *Zip1* deficiency was further explored. Because LYVE1 is a typical marker of LECs in initial lymphatics 45, LYVE1 was stained to distinguish initial and collecting lymphatics. In lymph nodes, the expression of Claudin-5 on LYVE1⁺ lymphatics was markedly elevated in *Zip1^-/-^* mice compared to *Zip1^+/+^* mice (**Figure S3A**). As for collecting lymphatics, whole-mount staining of mouse ear tissues was conducted. Initial lymphatics appeared as strong LYVE1 staining, while collecting vessels marked as Claudin-5^+^ accompanied with valvular structures. The results revealed that *Zip1* deficiency led to a robust upregulation of Claudin-5 across all compartments, including initial lymphatics, collecting vessels, and valve regions (**Figure S3B**).

### Lymphatic ZIP1 promotes trans-endothelial migration of tumor cells in a Claudin-5 dependent manner

To validate ZIP1-dependent regulation of lymphatic barrier function, the expression of ZIP1 in LECs was investigated. We analyzed the expression of *ZIP1/Zip1* in LECs using available single-cell sequencing data of both human and mouse pancreatic ductal adenocarcinoma (PDAC) with SCAR database 46. We picked up endothelial cells first and analyzed the co-expression of *ZIP1/Zip1* with *LYVE1/Lyve1*. In human/mouse PDAC 47, 48, a part of LECs was *ZIP1/Zip1* positive (**Figure S4, A and B**)*.* Next, we isolated CD31^+^PDPN^+^ LECs from LNs of *Zip1^+/+^* and *Zip1*^-/-^ mice and performed immunofluorescence staining (**Figure S4, C and D**). The results showed that ZIP1 was expressed in PROX1^+^ LECs (**Figure S4D**). *In vitro*, we constructed a LEC cell line SVEC4-10 with low expression of ZIP1 (SVEC4-10^kd^) using CRISPR/Cas9 technique and its control cell line (SVEC4-10^ctrl^) (**Figure 3A**). Zinc uptake was reduced in SVEC4-10^kd^ cells compared to the control (**Figure 3B**). To compare gene expression difference, we conducted transcriptomic analysis of gene expression in SVEC4-10^ctrl^ cells and SVEC4-10^kd^ cells. The results showed that *Zip1* (*Slc39a1*) gene was greatly expressed among zinc transporters in SVEC4-10 (**Figure S4E**), which was further confirmed by PCR (**Figure S4F**). Interestingly, *Zip1* gene knockdown induced downregulation of several zinc transporters (such as *Slc39a3, 11*), while induced compensatory upregulation of other zinc transporters (such as *Slc39a6, 10*) (**Figure S4G**), which is probably caused by zinc deficiency in *Zip1* knockdown cells. Simultaneously, zinc-associated *Znt* family genes and *Mt1*, *Mt2* were also altered (**Figure S4, H and I**). Noteworthily, as *Mt1/2* are Zn^2+^ inducible genes, it seems contradictory to observe their upregulation in *Zip1* knockdown cells (**Figure S4I**). This might be caused by the transport of other metals such as Cu²⁺, Mn²⁺ by upregulated ZIP6, 4, 10, 14) 49-51. Moreover, lymphatic marker gene *Vegfr3* (*Flt4*) and *Pdpn* were increased in SVEC4-10^kd^ cells compared to SVEC4-10^ctrl^ cells (**Figure S4J**). These results implied that ZIP1-dependent zinc regulation is crucial for zinc homeostasis and lymphatic phenotype regulation in LECs.

We performed a KEGG pathway enrichment analysis of the differentially expressed genes to investigate ZIP1-regulated pathways in LECs. Consistent with the above observation, “tight junction” was enriched (**Figure 3C; Figure S5, A and B**), indicating the regulation of intercellular junction by ZIP1. Because Claudin-5 is a typical tight junction protein expressed by both endothelial cells and lymphatic endothelial cells 15, 52, the expression of Claudin-5 in LEC with ZIP1 knockdown was examined. The results showed that the expression of Claudin-5 in SVEC4-10^kd^ was upregulated compared to the control (**Figure 3D**). Knockdown of ZIP1 by small RNA interference in SVEC4-10 upregulated the expression of Claudin-5 (**Figure 3, E and F**). The permeability of LEC monolayer to 70 kD FITC-Dextran was examined using transwell assay. The results showed that the leakiness of FITC-Dextran into the lower chamber across LEC monolayer of SVEC4-10^kd^ cells lower than that of SVEC4-10^ctrl^ cells (**Figure 3, G and H**). The paracellular tightness of SVEC4-10^ctrl^ or SVEC4-10^kd^ confluent monolayers was detected based on cellular electrical impedance. The results showed that SVEC4-10^kd^ cells formed a monolayer along with increased intercellular tightness (**Figure 3I**). These results suggest that the expression of ZIP1 by LECs induces an opening of lymphatic endothelial junctions.

To examine the ability of tumor cells to migrate across endothelial monolayers formed by SVEC4-10^ctrl^ or SVEC4-10^kd^ cells, we performed trans-endothelial migration assay. Pan02 cells labeled with CMFDA were added onto endothelial cell monolayers in the upper chamber and allowed to migrate across the cell monolayers. After removing unmigrated tumor cells in the upper chamber, migrated tumor cells with green fluorescence were photographed with confocal microscopy. The results showed that SVEC4-10^kd^ cell monolayer limited the cross of tumor cells compared to SVEC4-10^ctrl^ cell monolayer (**Figure 3, J and K**), indicating that ZIP1 expression in LECs facilitates trans-endothelial migration of pancreatic cancer cells. In addition, Claudin-5 was further knockdown in SVEC4-10^kd^ cells, which recovered the ability to facilitate trans-endothelial migration of Pan02 cells (**Figure 3L-N**), suggesting that lymphatic ZIP1 promotes trans-endothelial migration of tumor cells in a Claudin-5 dependent manner. In addition, *ZIP1* was knockdown in primary human LEC (hLEC) (**Figure 3O-Q**). Knockdown of *ZIP1* reduced the permeability of hLEC monolayer to FITC-Dextran and restricted trans-endothelial migration of PANC-1 across hLEC monolayer.

### ZIP1 enhances lymphatic glycolysis to decrease the expression of Claudin-5

To investigate how ZIP1 regulates lymphatic intercellular junction, we conducted GO analysis of altered genes between SVEC4-10^ctrl^ cells and SVEC4-10^kd^. The results revealed that metabolic pathways were significantly enriched (**Figure 4A; Figure S5, C and D**). Then, we explored the effect of ZIP1 on the glycolysis and mitochondrial respiration of LECs by Seahorse metabolic analyzer. The results showed that the glycolysis was inhibited in SVEC4-10^kd^ cells, while mitochondrial respiration was not significantly affected, compared to SVEC4-10^ctrl^ cells (**Figure 4B-D**), suggesting that ZIP1 sustains high glycolysis in LECs. Previous studies have demonstrated that elevated cytosolic zinc bolsters glycolysis via activation of the AKT-mTOR-S6K signaling cascade 53-55. Consistent with this, zinc treatment activated the AKT-mTOR-S6K pathway in SVEC4-10 cells (**Figure S6A**). In contrast, the phosphorylation levels within this pathway were markedly attenuated in SVEC4-10^kd^ cells compared to the control cells (**Figure S6B**). Moreover, ZIP1 knockdown reduced the expression of GLUT-1 and p-PFKFB2 (**Figure 4, E and F**), indicating that ZIP1 affects glucose uptake and downstream catalytic process. Consistently, SVEC4-10^kd^ cells showed less glucose uptake than SVEC4-10^ctrl^ cells (**Figure 4, G and H**). In addition, glycolysis was also decreased in primary human LECs with si-*ZIP1* (**Figure 4, I and J**). Interestingly, blocking glycolysis by 2-DG, a glucose analog, upregulated Claudin-5 in SVEC4-10 (**Figure 4K**). In contrast, lactic acid, as the main product of glycolysis, inhibited the expression of Claudin-5 (**Figure 4L**). Treatment with MG132, a proteasomal inhibitor, did not significantly alter Claudin-5 expression. However, administration of Bafilomycin A1, a lysosomal inhibitor, markedly upregulated Claudin-5 levels (**Figure S7A**). Furthermore, Bafilomycin A1 treatment abrogated the lactate-induced downregulation of Claudin-5 (**Figure S7B**). These results indicate that Claudin-5 is primarily degraded via the autophagy-lysosomal system, a process that is facilitated by lactate, the principal metabolite of glycolysis. When treated with lactic acid, the permeability of SVEC4-10 cell monolayer to FITC-Dextran and trans-endothelial migration of Pan02 tumor cells across LEC monolayer were increased (**Figure 4, M and N**). When treated with 2-DG, trans-endothelial migration of Pan02 tumor cells across LEC monolayer was reduced (**Figure 4O**). Injection of 2-DG at the mouse ear restricted the lymph draining of FITC-Dextran to the cervical LNs (**Figure 4, P and Q**). However, Western blot analysis revealed that short-term exogenous Zn^2+^ supplementation alone had a limited effect on glycolysis and Claudin-5 expression in SVEC4-10^kd^ cells. In contrast, combined treatment with Zn^2+^ and PYT (a zinc ionophore that rapidly transports extracellular Zn^2+^ into the cytosol independent of zinc transporters, resulting in a sharp increase in labile intracellular zinc) significantly rescued the alterations in glycolytic activity and Claudin-5 levels induced by ZIP1 deficiency (**Figure S8, A and B**). These findings demonstrate that intracellular zinc concentration is a critical determinant of lymphatic endothelial barrier function, and that ZIP1, serving as a zinc transporter, plays a pivotal role in this regulatory process.

### Tumor-derived S100A4 upregulates ZIP1 in LECs

Whether lymphatic ZIP1 was dysregulated in tumor was investigated. The expression of ZIP1 in lymphatic vessels from normal skin and Pan02 tumor was compared, and the results showed that lymphatic vessels in Pan02 tumor expressed higher level of ZIP1 than those in normal skin (**Figure 5, A and B**). When SVEC4-10 were treated with tumor supernatants, ZIP1 was upregulated, the permeability of endothelial cell monolayer to FITC-Dextran increased, and the cellular impedance decreased (**Figure 5, C-E**). Moreover, trans-endothelial migration of Pan02 tumor cells was enhanced by the tumor supernatant treatment (**Figure 5F**). In contrast, the tumor supernatant treatment did not significantly increase trans-endothelial migration of Pan02 tumor cells across SVEC4-10^kd^ cell monolayers (**Figure 5G**), suggesting that ZIP1 is essential for the regulation of lymphatic barrier function by tumor-derived factors.

To screen factors that regulated ZIP1, we collected gene expression data of a pancreatic cancer cell line PANC-1 from the Expression Atlas database (www.ebi.ac.uk), and analyzed the expression of secretory protein genes, which showed that PANC-1 highly expressed genes including *S100A4*, *TGFB1* and *VEGFC* (**Figure 5H**). We validated the expression of these secretory protein genes by RT-PCR in Pan02 cells and *S100a4*, C*sf2*, *Tgfb1* and *Vegfc* were expressed by tumor cells (**Figure 5I**). Our previous studies suggest that S100A4 upregulates ZIP1 in tumor-associated fibroblasts of lung cancer, and intracellular S100A4 enhances glycolysis to promote lymphatic sprouting in melanoma 29, 56. The results showed that S100A4 protein was expressed by Pan02 tumor cells and could be further upregulated by chemotherapeutic drug paclitaxel but not gemcitabine treatment (**Figure 5, J and K**). Therefore, we examined whether S100A4 could regulate ZIP1 in LECs, as well as lymphatic barrier function. The results showed that neutralization of S100A4 reduced tumor supernatants-induced ZIP1 expression in SVEC4-10 cells (**Figure 5L**). S100A4 stimulation directly increased the expression of ZIP1 in a dose dependent manner (**Figure 5M**). Downstream signaling mediators, including NF-κB (p65), AKT, ERK, and p38, were activated upon S100A4 stimulation, and their respective inhibitors attenuated the S100A4-mediated upregulation of ZIP1 (**Figure S9, A and B**). Given that RAGE and TLR4 serve as receptors for S100A4, we further demonstrated that the S100A4-induced upregulation of ZIP1 was diminished by the RAGE inhibitor FPS-ZM1 and the TLR4 inhibitor TLR4-IN-C34 (**Figure S9C**). These results indicate that S100A4 binds to RAGE/TLR4 to activate the NF-κB (p65), AKT, ERK, and p38 signaling cascades, thereby promoting ZIP1 expression in SVEC4-10 cells. When S100A4 was neutralized, the impairment of cellular impedance and the increase of cellular permeability to FITC-Dextran caused by tumor supernatants were reversed (**Figure 5, N and O**). The addition of S100A4 increases the permeability of SVEC4-10 cell monolayers to 70 kD FITC-Dextran, and decreased cellular integrity of LECs (**Figure 5, P and Q**). Intriguingly, S100A4 treatment did not significantly increase the permeability of SVEC4-10^kd^ cell monolayers to FITC-Dextran (**Figure 5R**), suggesting that ZIP1 is essential for regulation of lymphatic barrier function by S100A4. Furthermore, S100A4 was knockout (KO) in Pan02-GFP-Luc cells and transplanted into the mouse footpads. Compared to the mock cancer cells transplanted to *Zip1^+/+^* mice, S100A4-KO cancer cells rarely metastasized into the LNs of both *Zip1^+/+^* and *Zip1^-/-^* mice (**Figure 5, S and T**). These results suggest that ZIP1-dependent lymphatic adaptations could be hijacked by cancer cells through producing S100A4.

### Lymphatic-specific *Zip1* knockout in mice improves lymphatic barrier function leading to reduced LN metastasis

To validate that ZIP1-mediated metabolic and junctional adaptations in LECs affected LN metastasis, we constructed a conditional knockout mouse (*Zip1^ΔLyve1^, Zip1^flox/ flox^*; *Lyve1-Cre+*), in which *Zip1* was deleted in LYVE1^+^ lymphatic vessels (**Figure 6A**, **Figure S10, A and B**). To validate the deletion of *Zip1,* LYVE1-positive cells in LNs were sorted, and the expression of *Zip1* was detected by RT-PCR. The results showed that *Zip1* was effectively suppressed in these cells (**Figure S10, C and D**). Furthermore, we evaluated whether the LEC-specific *Zip1* knockout affected the density of lymphatic vessels in naïve mice. The results showed that no obvious differences in lymphatic vessel density were observed in the skin between *Zip1^flox/flox^* mice and *Zip1^ΔLyve1^* mice, consistent with the observation in *Zip1^+/+^* and *Zip1^-/-^* mice (**Figure S10, E and F**). However, Claudin-5 expression on LYVE1^+^ LECs was also strengthened in the normal skin of *Zip1^-/-^* and* Zip1^ΔLyve1^* mice compared to that of *Zip1^flox/flox^* mice (**Figure S10G**). The results showed that lymph draining of FITC-Dextran was decreased in *Zip1^ΔLyve1^* mice, compared to the control *Zip1^flox/flox^* mice (**Figure 6, B and C**). These results suggest that ZIP1 in LECs control lymphatic barrier function.

Pan02-GFP-Luc cells were then injected into the footpads of *Zip1^flox/flox^* mice and *Zip1^ΔLyve1^* mice to examine tumor lymphatic changes and lymphatic metastasis. The lymphatic vessel density in tumors was not significantly different between *Zip1^flox/flox^* mice and *Zip1^ΔLyve1^* mice (**Figure 6D**). However, Claudin-5 expression on LYVE1^+^ LECs was drastically enhanced in *Zip1^ΔLyve1^* mice compared to *Zip1^flox/flox^* mice (**Figure 6E**). Consistently, tumor LN metastasis was reduced in *Zip1^ΔLyve1^* mice compared to *Zip1^flox/flox^* mice, while tumor growth was similar between *Zip1^flox/flox^* mice and *Zip1^ΔLyve1^* mice (**Figure 6F-H, Figure S10H**). Tumor cell metastasis in LNs was confirmed by HE staining and flow cytometry (**Figure 6I-K**). Distant lung metastasis in these two mouse strains was not significantly different (**Figure S10, I and J**). Tight junction protein expression was then detected on blood vessels. The results showed that Claudin-5 expression on tumor endothelial cells (ECs) was upregulated in *Zip1^-/-^* mice, whereas ZO-1 levels remained unchanged (**Figure S11, A and B**). In contrast, neither Claudin-5 nor ZO-1 expression was significantly altered in lymphatic-specific knockout *Zip1^flox/flox^*; *Prox1-Cre+* mice (**Figure S11, C and D**). These results suggest that ZIP1 also affects endothelial tight junctions, which might contribute to lung metastasis.

To further validate the above observations, we constructed an alternative conditional knockout mouse, in which *Zip1* was deleted in PROX1^+^ lymphatic vessels (**Figure 6L**). LN metastasis of Pan02-GFP-Luc in these mice was examined. The results showed that one or both allele deletion of *Zip1* in Prox1^+^ cells (*Zip1^flox/^*^+^; *Prox1-Cre+, Zip1^flox/flox^*; *Prox1-Cre+*) was efficient to reduce LN metastasis of cancer cells (**Figure 6, L and M**). Immunofluorescence staining of tumor tissue sections revealed that GLUT-1 expression on LYVE1^+^ LECs was significantly reduced in tumors from *Zip1^flox/flox^*; *Prox1-Cre+* mice compared to controls (**Figure S12A**), implying that ZIP1 deletion induced the metabolic alterations in lymphatic endothelial cells *in vivo*. In summary, ZIP1-dependent lymphatic adaptations facilitate LN metastasis of pancreatic cancer.

### ZIP1-expressing lymphatics are associated with LN metastasis of human PDAC

To explore the association of ZIP1-expressing lymphatics with LN metastasis of human PDAC, a tumor tissue array (TMA) containing 88 tumor tissues from PDAC patients was adopted, and multiplex staining of LYVE1, ZIP1 and Claudin-5 was performed. The expression of ZIP1 and Claudin-5 on LYVE1^+^ lymphatics was detected (**Figure 7A**). And a negative correlation (R = -0.390, P = 0.006) of ZIP1 and Claudin-5 on LYVE1^+^ lymphatics was observed (**Figure 7B**), corresponding to the above observation in mouse tumor tissues. To investigat the association of lymphatics with clinical parameters, the area of LYVE1^+^ LEC, and the area of ZIP1^+^LYVE1^+^ LEC in each tumor section were evaluated and distinguished into low and high groups. The results showed that LEC^high^ was associated with more LN metastasis and the death of patients (**Figure 7C, Table S1**). ZIP1^+^ LEC^high^ was associated with more LN metastasis and aggressive tumor stage (**Figure 7D, Table S2**). Survival analysis showed that LEC^high^ and ZIP1^+^ LEC^high^ predicted poor overall survival and progression-free survival (**Figure 7E-H**). These results suggest that ZIP1-expressing lymphatics are associated with LN metastasis of human PDAC and predict poor survival of patients.

## Discussion

A communication between tumor cells and lymphatic endothelial cells is required for the development of lymphatic metastasis of pancreatic cancer 8, 12. Lymphatic vessels at the capillary level already bear molecular traits and anatomical structures that control cell in and out. In response to tumor stimuli, a further coordination of lymphatic vessels occurs to accept the intravasation of tumor cells 8. However, the mechanisms by which LECs undergo adaptations in the process of LN metastasis of pancreatic cancer remain largely unknown. Zinc dyshomeostasis has been observed in multiple tumors, including pancreatic cancer, with its impact on lymphatic remodeling unclear. In this study, we find that ZIP1 in LECs control the opening of lymphatic barrier, which is dependent on glycolysis. Tumor-derived S100A4 could upregulate ZIP1 in LECs, inducing further opening of lymphatic barrier and promoting trans-endothelial migration of tumor cells. Lymphatic-specific knockout of *Zip1* dramatically improves lymphatic barrier function and inhibits tumor LN metastasis in mice. Importantly, ZIP1^+^ lymphatics are associated with LN metastasis of human PDAC and poor survival of patients. Our findings reveal that ZIP1-mediated lymphatic metabolic and junctional adaptations accelerate LN metastasis of pancreatic cancer, providing special insights into LN metastasis of pancreatic cancer, and forming a unique basis for developing strategy to treat pancreatic cancer metastasis.

Lymphatic adaptations in LN metastasis of pancreatic cancer are dependent on ZIP1. Zinc signaling is crucial for the maintenance of intestinal epithelial barrier function in normal and disease conditions 57. In contrast, zinc accumulation in ischemic tissue contributes to blood-brain barrier (BBB) disruption and ZnT3 knockout in neurons markedly reduces zinc in extracellular fluid and BBB permeability 58. In this study, we demonstrated that lymphatic barrier function is largely improved in *Zip1*^-/-^ mice and *Zip1* lymphatic-specific knockout mice, compared to the control mice, indicating ZIP1 physiologically controls proper opening of lymphatic barrier. *In vitro* knockdown of *Zip1* in LECs directly decreases Zn^2+^ uptake and glycolysis, promotes the expression of Claudin-5 and reduces the permeability of LEC monolayer. When inhibiting glycolysis by 2-DG, lymph draining is restricted. These results suggest that ZIP1 sustains glycolysis to control the opening of lymphatic endothelial junctions. Metabolism pathway activation including glycolysis and fatty acid β-oxidation regulates lymphatic functions 21. Our previous study show that glycolysis promotes lymph sprouting 36. In this study, we find that ZIP1 expression enhances glycolysis, which produces lactic acid and boosts Claudin-5 degradation. In brief, LECs in tumor enhance the capability of zinc uptake by upregulating ZIP1, which promotes glycolysis and subsequently impairs Claudin-5. Nevertheless, measuring the absolute zinc contents in tissues, including lymph nodes, will be helpful for the understanding of lymphoid Zn^2+^ homeostasis.

Cancer cells hijack ZIP1-dependent controlling of lymphatic function in LN metastasis. Instead of playing as a passive route for tumor cell trafficking, lymphatic vessels experience dynamic changes to facilitate lymphatic metastasis 9. We hypothesize that the chaotic tumor niche imposes selection stresses on LECs to trigger their adaptations that coordinately facilitate lymphatic metastasis. Common stresses in tumor might include hypoxia, mechanical stiffness, inflammation and fibrosis 59. Here, we postulated that zinc dyshomeostasis is a hallmark of cancer and a potential stress that drives tumor malignant progression, including lymphatic metastasis 60. High proliferating activity of cancer cells would request a timely and adequately zinc supply for use, which subsequently may cause zinc deficiency in the tumor microenvironment. Many studies including ours have demonstrated that zinc dyshomeostasis is a common phenomenon in various tumors, including pancreatic cancer, and has important impact on tumor progression 23, 29, 60, 61. Therefore, we propose that LECs would adapt to zinc dyshomeostasis and then contribute to lymphatic metastasis. In this study, our results reveal a ZIP1-dependent regulation of lymphatic functions. In tumor setting, tumor-derived factors, specifically S100A4 can further upregulate ZIP1 in LECs, thereby opening lymphatic intercellular junctions and promoting trans-endothelial migration of tumor cells. In consistence, previous study demonstrates that lymphatic barrier limits tumor intravasation 8. In pancreatic cancer, the expression of S100A4 is associated with drug resistance, differentiation, metastasis and clinical outcome 62, 63. These observations support the idea that LECs acquire adaptations to zinc dyshomeostasis in tumor, and cancer cells hijack ZIP1-dependent lymphatic tuning, leading to LN metastasis. Considering tumor cells shift to increase lipid metabolism in lymphatic metastasis 64, it is interesting to observe a glycolytic shift in LECs, which exquisitely avoiding metabolic competition between tumor cells and LECs. Our study shows that pancreatic cancer cells also secrete TGFβ and VEGFC. Whether these factors are involved in lymphatic regulation merits further investigation. Additionally, while our model recapitulates metastasis-to-metastasis of pancreatic cancer, orthotopic pancreatic cancer models that simulate primary-to-metastasis are needed in future study to validate and extend our key findings.

ZIP1-dependent lymphatic adaptations may be explored for clinical diagnosis and treatment of pancreatic cancer. LN metastasis is prevalent in patients with advanced pancreatic cancer, as well as in patients with relapse. Our study suggests that ZIP1^+^ LEC^high^ predicts severe LN metastasis of pancreatic cancer, and poor survival of patients. Lymphatic ZIP1 expression, glycolysis and Claudin-5 expression might be explored as diagnostic markers of LN metastasis of pancreatic cancer. Our previous study 36 and current work demonstrate that S100A4 plays a crucial role in lymphatic remodeling that facilitates LN metastasis. In patients with pancreatic cancer, increased S100A4 expression is associated with the high tumor-node-metastasis stages 65. Therefore, targeting S100A4 is probably beneficial for patients with pancreatic cancer. In conclusion, our study suggests a mechanism of ZIP1-dependent lymphatic adaptations that lead to LN metastasis of PDAC, which provides potential therapeutic targets for clinical treatment of pancreatic cancer metastasis.

## Supplementary Material

Supplementary figures and tables.

## Figures and Tables

**Figure 1:**
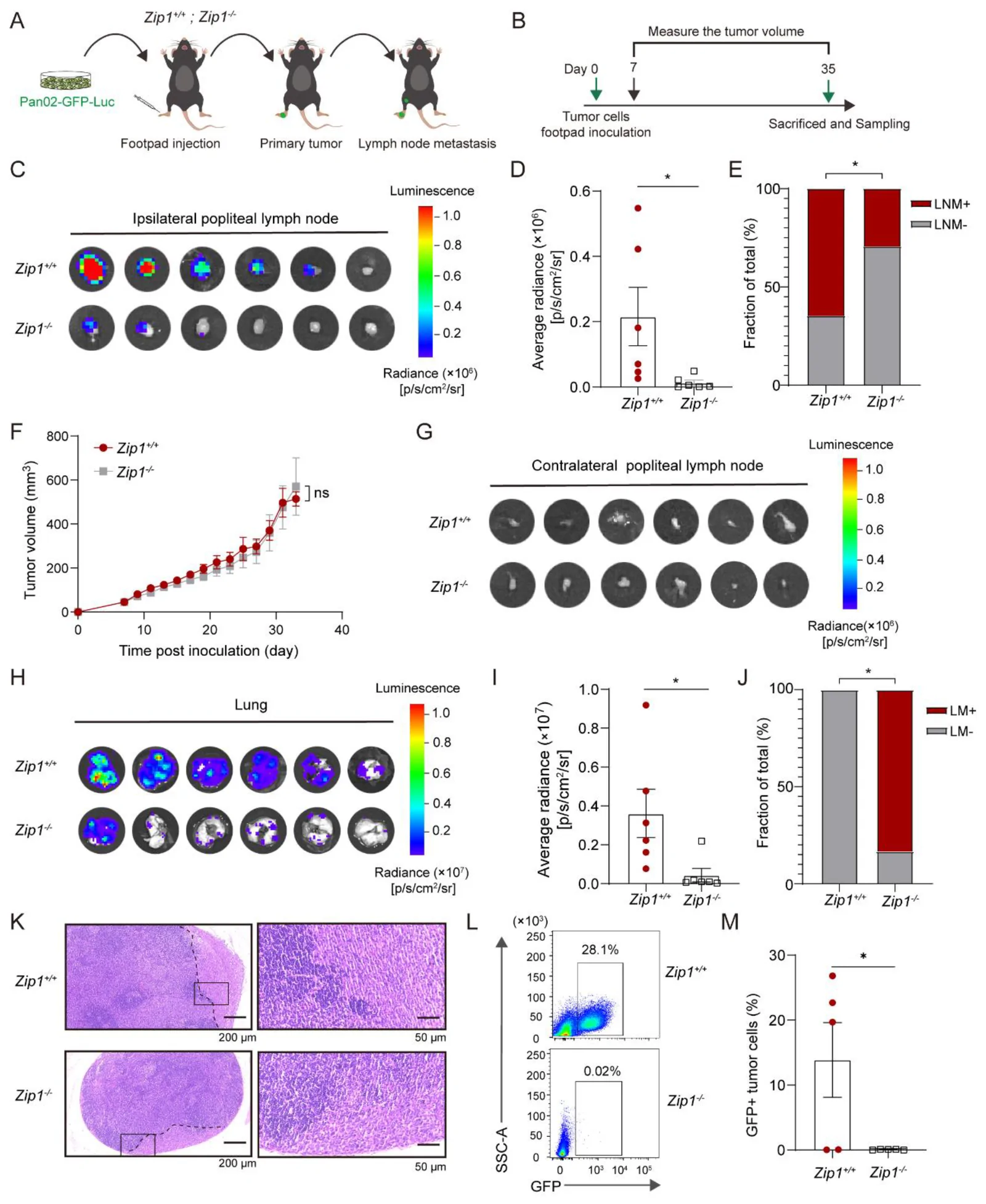
** Host ZIP1 deficiency reduces LN metastasis of pancreatic cancer. (A, B)** Schematic representation of animal experiments of popliteal LN metastasis. Pan02-GFP-Luc cells were inoculated into the footpad of *Zip1^+/+^* and *Zip1^-/-^* mice. After 35 days, mice were sacrificed and tumor metastasis was examined. **(C)** Metastasis in the popliteal LNs ipsilateral to the footpad tumor from *Zip1^+/+^* and *Zip1^-/-^* mice detected by bioluminescence (n = 6 for each group). **(D)** Quantification of bioluminescence intensity of LN metastasis shown above.** (E)** Quantification of LNs with metastasis (LNM) in three independent animal experiments (n = 3 × 6 = 18 for each group). **(F)** Primary tumor growth of Pan02-GFP-Luc cells monitored over time.** (G)** Metastasis in the popliteal LNs contralateral to the footpad tumor from *Zip1^+/+^* and *Zip1^-/-^* mice detected by bioluminescence. **(H)** Lung metastasis in *Zip1^+/+^* and *Zip1^-/-^* mice detected by bioluminescence.** (I)** Quantification of bioluminescence intensity of lung metastasis shown above. **(J)** Quantification of Lungs with metastasis (LM) in (H) (n = 6 for each group).** (K)** Representative HE staining of LN metastasis. Scale bar, 200 μm (left) and 50 μm (right).** (L)** Flow cytometry analysis of GFP^+^ tumor cells in ipsilateral popliteal lymph nodes. **(M)** Quantification of GFP^+^ cells in indicated groups as in **(L)**. *p < 0.05**.** Data are representative of at least three independent experiments.

**Figure 2:**
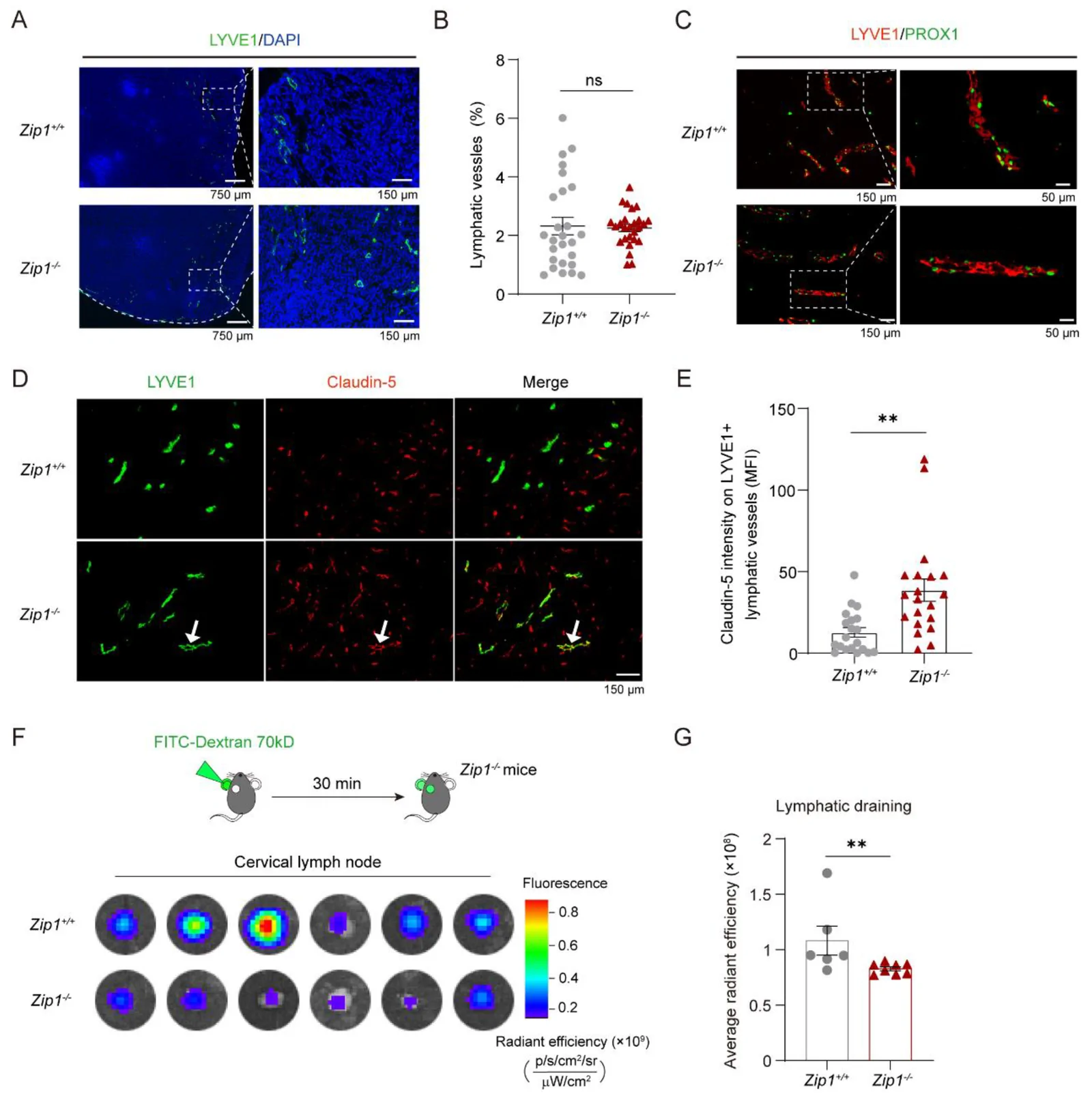
** Intercellular junctions of lymphatic vessels in tumors are enhanced in *Zip1^-/-^* mice. (A)** Representative images showing LYVE1^+^ lymphatic vessels in Pan02-GFP-Luc tumor sections from *Zip1^+/+^* and *Zip1^-/-^* mice. Scale bar, 750 μm (left) and 150 μm (right). **(B)** Quantification of LYVE1^+^ vessel area per field in tumors from *Zip1^+/+^* and *Zip1^-/-^* mice. (n = 26 fields per group).** (C)** Representative images showing LYVE1 and PROX1 immunostaining in Pan02-GFP-Luc tumor sections from *Zip1^+/+^* and *Zip1^-/-^* mice. Higher-magnification images showing the colocalization of LYVE1^+^ and PROX1^+^ LECs. Scale bar, 150 μm (left) and 50 μm (right). **(D)** Representative images showing LYVE1 and Claudin-5 immunostaining in Pan02-GFP-Luc tumor sections from *Zip1^+/+^* and *Zip1^-/-^* mice. Scale bar, 150 μm. **(E)** Quantification of Claudin-5 fluorescence intensity on LYVE1^+^ lymphatics in **(D)** (n = 20 fields per group).** (F)** Representative images showing draining of FITC-Dextran into cervical lymph nodes after injection at the ear of indicated mice. **(G)** Quantification of FITC-Dextran fluorescence in cervical lymph nodes of indicated groups (n = 6 for *Zip1^+/+^*; n = 8 for *Zip1^-/-^*). *p < 0.05, **p < 0.01.

**Figure 3:**
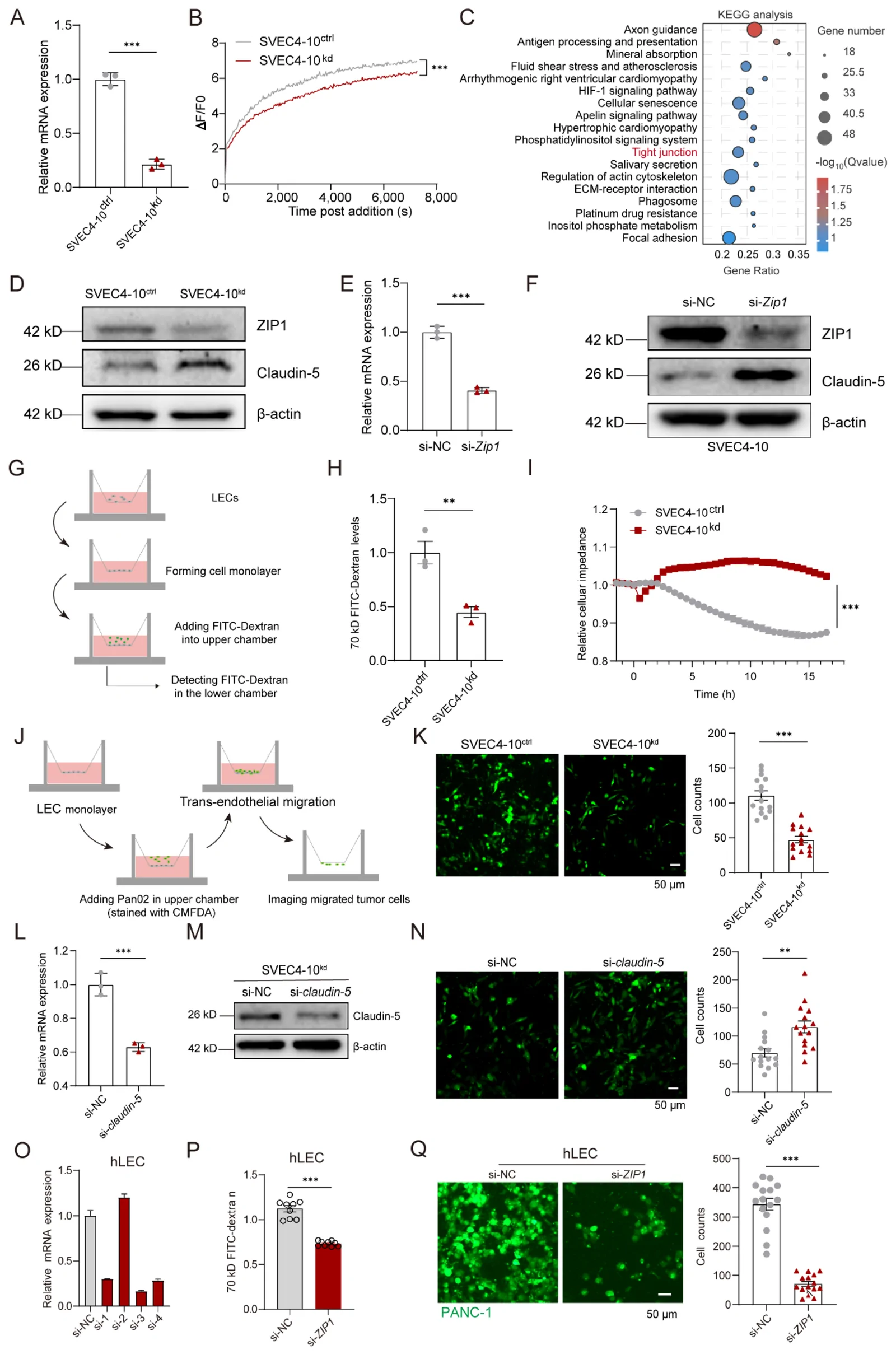
** ZIP1 regulates trans-endothelial migration of tumor cells in a Claudin-5 dependent manner. (A)** The mRNA expression of *Zip1* in SVEC4-10^ctrl^ and SVEC4-10^kd^ cells. **(B)** Zn^2+^ uptake by SVEC4-10^ctrl^ and SVEC4-10^kd^ cells (n = 3 per group). ZnCl_2_, 30 μM. **(C)** KEGG analysis of differential expressed genes (including both upregulated and downregulated genes) between SVEC4-10^ctrl^ and SVEC4-10^kd^ cells showing representative enriched pathways.** (D)** The expression of ZIP1 and Claudin-5 in SVEC4-10^ctrl^ and SVEC4-10^kd^ cells. **(E)** The mRNA expression of *Zip1* in SVEC4-10 with RNA interference targeting *Zip1* (si-*Zip1*) (n = 3 per group). si-NC: negative control. **(F)** The expression of ZIP1 and Claudin-5 in SVEC4-10 with si-*Zip1*.** (G)** Schematic showing the permeation assay for LECs. **(H)** The permeability of indicated LEC monolayer for 70 kD FITC-Dextran.** (I)** Paracellular tightness of indicated LEC monolayer evaluated by relative cellular impedance.** (J)** Schematic illustration of trans-endothelial migration assay.** (K)** Trans-endothelial migration of Pan02 across indicated LEC monolayer after 24 h. (n = 15 fields per group). Scale bar, 50 μm. **(L)** The mRNA expression of *Claudin-5* in SVEC4-10^kd^ with RNA interference targeting *Claudin-5* (si-*Claudin-5*) (n = 3 per group). **(M)** The expression of claudin-5 in SVEC4-10^kd^ with si-*Claudin-5*.** (N)** Trans-endothelial migration of Pan02 across indicated LEC monolayer after 16 h (n = 15 fields per group). **(O)** Knockdown of *ZIP1* (si-1, 2, 3, 4) in hLEC. si-3 was used in following study.** (P)** The permeability of indicated hLEC monolayer for 70 kD FITC-Dextran.** (Q)** Trans-endothelial migration of PANC-1 across indicated hLEC monolayer after 24 h. (n = 15 fields per group). Scale bar, 50 μm. **p < 0.01, ***p < 0.001.

**Figure 4:**
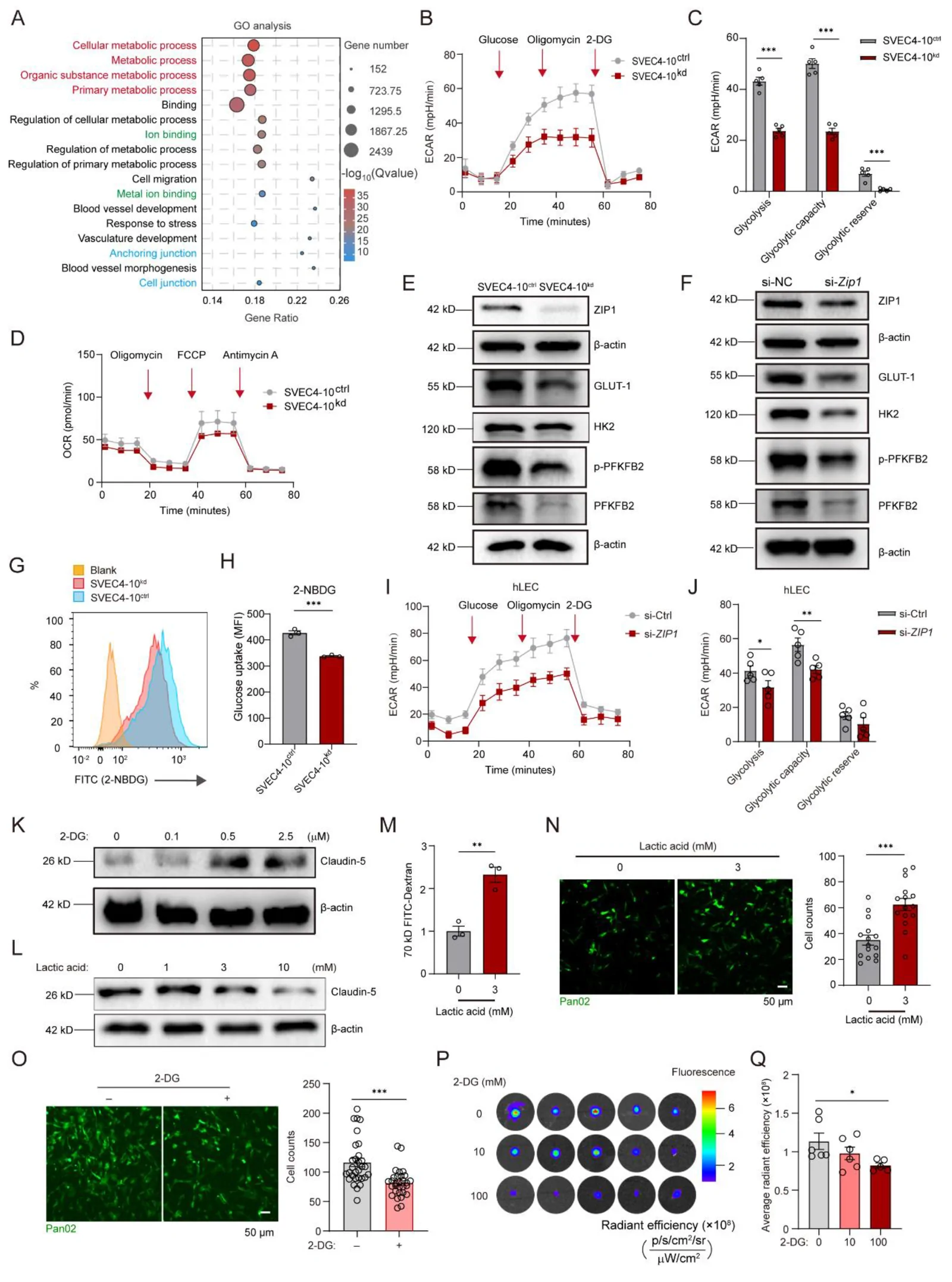
** ZIP1 promotes glycolysis to decrease the expression of Claudin-5. (A)** GO analysis of differential expressed genes (including both upregulated and downregulated genes) between SVEC4-10^ctrl^ and SVEC4-10^kd^ cells showing representative enriched pathways.** (B)** Extracellular acidification rate (ECAR) of SVEC4-10^ctrl^ and SVEC4-10^kd^ cells after sequential injection of glucose, oligomycin, and 2-DG. **(C)** Glycolysis, glycolytic capacity, and glycolysis reserve capacity for SVEC4-10^ctrl^ and SVEC4-10^kd^ cells (n ≥ 5 per group).** (D)** Oxygen consumption rate (OCR) of SVEC4-10^ctrl^ and SVEC4-10^kd^ cells after sequential injection of oligomycin, FCCP and antimycin A.** (E)** The expression of ZIP1, GLUT-1, HK2, p-PFKFB2, PFKFB2 in SVEC4-10^ctrl^ and SVEC4-10^kd^ cells. **(F)** The expression of ZIP1, GLUT-1, HK2, p-PFKFB2, PFKFB2 in SVEC4-10 with si-*Zip1*. **(G)** Representative flow cytometry results showing 2-NBDG uptake by SVEC4-10^ctrl^ and SVEC4-10^kd^ cells.** (H)** Quantification of mean fluorescence intensity (MFI) of 2-NBDG in indicated cells. **(I)** ECAR of indicated hLEC cells after sequential injection of glucose, oligomycin, and 2-DG. **(J)** Glycolysis, glycolytic capacity, and glycolysis reserve capacity for indicated hLEC cells (n ≥ 5 per group). **(K)** The expression of Claudin-5 in SVEC4-10 cells with 2-DG stimulation at different doses. **(L)** The expression of Claudin-5 in SVEC4-10 cells with lactic acid stimulation at different doses.** (M)** The permeability of SVEC monolayer with lactic acid treatment for 70 kD FITC-Dextran.** (N)** Trans-endothelial migration of Pan02 across SVEC monolayer with lactic acid treatment after 16 h (n = 15 fields per group). Scale bar, 50 μm. **(O)** Trans-endothelial migration of Pan02 across SVEC monolayer with 2-DG (5 μM) treatment after 24 h (n = 30 fields per group). Scale bar, 50 μm.** (P)** Representative images showing draining of FITC-Dextran into cervical lymph nodes. Injected 1 μL 2-DG (0, 10, 100 mM) 12 h ahead and following 70 kD FITC-Dextran at the mouse ear. After 30 min, ipsilateral cervical lymph nodes were collected and examined. **(Q)** Quantification of FITC-Dextran fluorescence in cervical lymph nodes of indicated groups (n ≥ 5 per group). *p < 0.05, **p < 0.01, ***p < 0.001.

**Figure 5:**
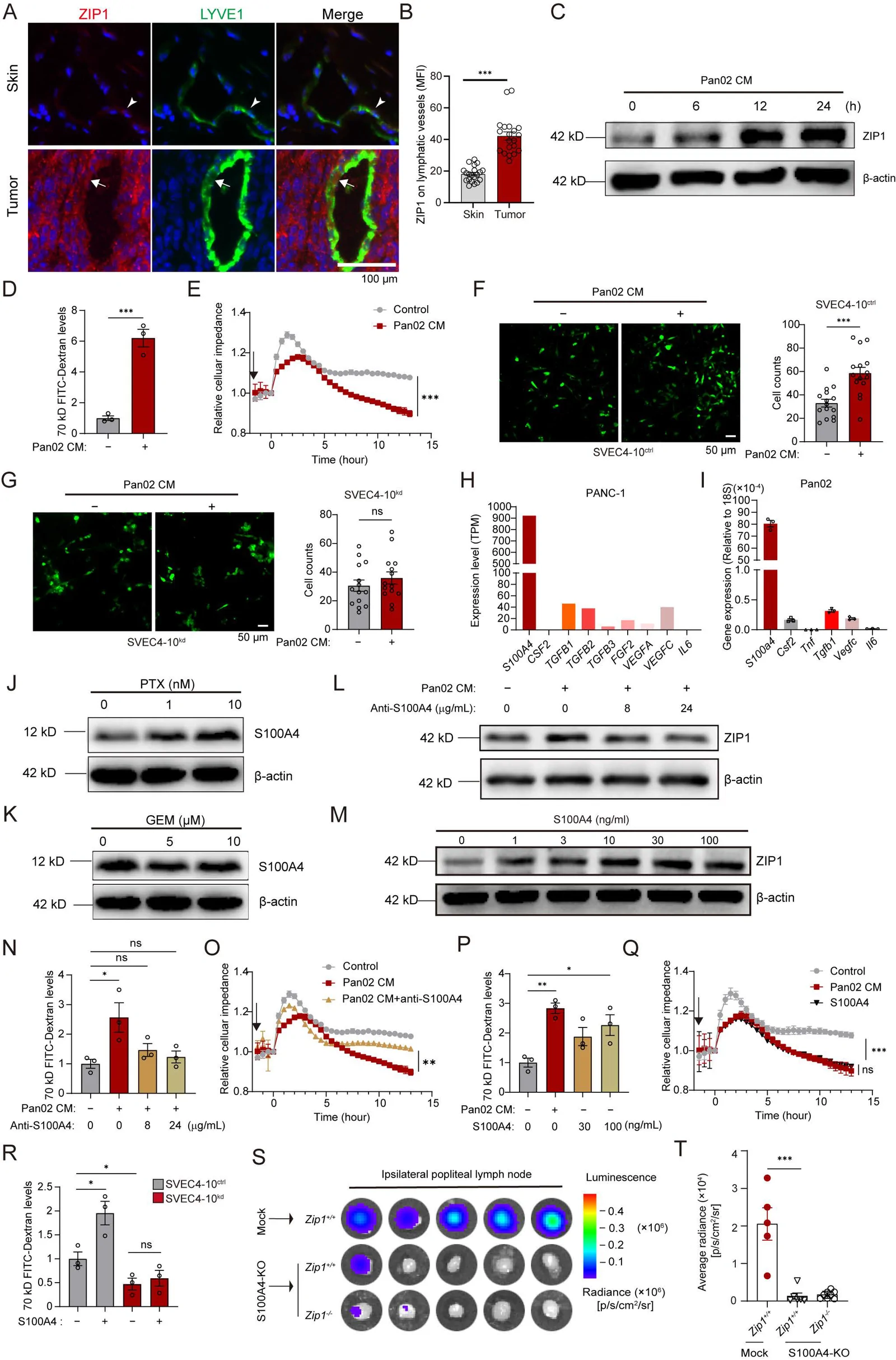
** Tumor-derived S100A4 regulates the expression of ZIP1 on lymphatic endothelial cells. (A)** Representative images showing LYVE1 and ZIP1 staining of normal skin and tumor tissue sections. Scale bar, 100 µm. **(B)** Quantification of mean fluorescence intensity (MFI) of ZIP1 on LYVE1^+^ lymphatics in **(A)** (n = 20 fields per group).** (C)** The expression of ZIP1 in SVEC4-10 cells stimulated by Pan02 culture medium (CM). **(D)** The permeability of SVEC4-10 monolayer for FITC-Dextran stimulated by Pan02 CM.** (E)** The paracellular tightness of SVEC4-10 monolayer stimulated by Pan02 CM.** (F)** Trans-endothelial migration of Pan02 (stained with CMFDA) across SVEC4-10 monolayer stimulated by Pan02 CM (n = 15 fields per group). Scale bar, 50 μm. **(G)** Trans-endothelial migration of Pan02 across SVEC4-10^kd^ monolayer stimulated by Pan02 CM (n = 15 fields per group). Scale bar, 50 μm. **(H)** The expression of cytokines by PANC-1 analyzed with Expression Atlas, a database.** (I)** The expression of cytokines by Pan02 analyzed by RT-PCR (n = 3 per group).** (J, K)** The expression of S100A4 by Pan02 cells treated with paclitaxel (PTX) **(J)** and gemcitabine (GEM) **(K)**. **(L)** The expression of ZIP1 in SVEC4-10 cells with indicated treatments.** (M)** The expression of ZIP1 in SVEC4-10 cells with S100A4 at indicated doses.** (N)** The permeability of SVEC4-10 monolayer for FITC-Dextran with indicated stimulation.** (O)** The paracellular tightness of SVEC4-10 monolayer with indicated stimulation.** (P)** The permeability of SVEC4-10 monolayer for FITC-Dextran with indicated stimulation. **(Q)** The paracellular tightness of SVEC4-10 monolayer stimulated by Pan02 CM or S100A4 (30 ng/mL).** (R)** The permeability of SVEC4-10^ctrl^ and SVEC4-10^kd^ monolayer for FITC-Dextran stimulated by S100A4. **(S)** Representative images showing metastasis of Pan02-GFP-luc with S100A4 knockout (KO) and the mock cells in the popliteal LNs from *Zip1^+/+^* or *Zip1^-/-^* mice detected by bioluminescence (n ≥ 5 per group). **(T)** Quantification of bioluminescence intensity of LN metastasis of indicated groups. *p < 0.05, **p < 0.01, ***p < 0.001.

**Figure 6:**
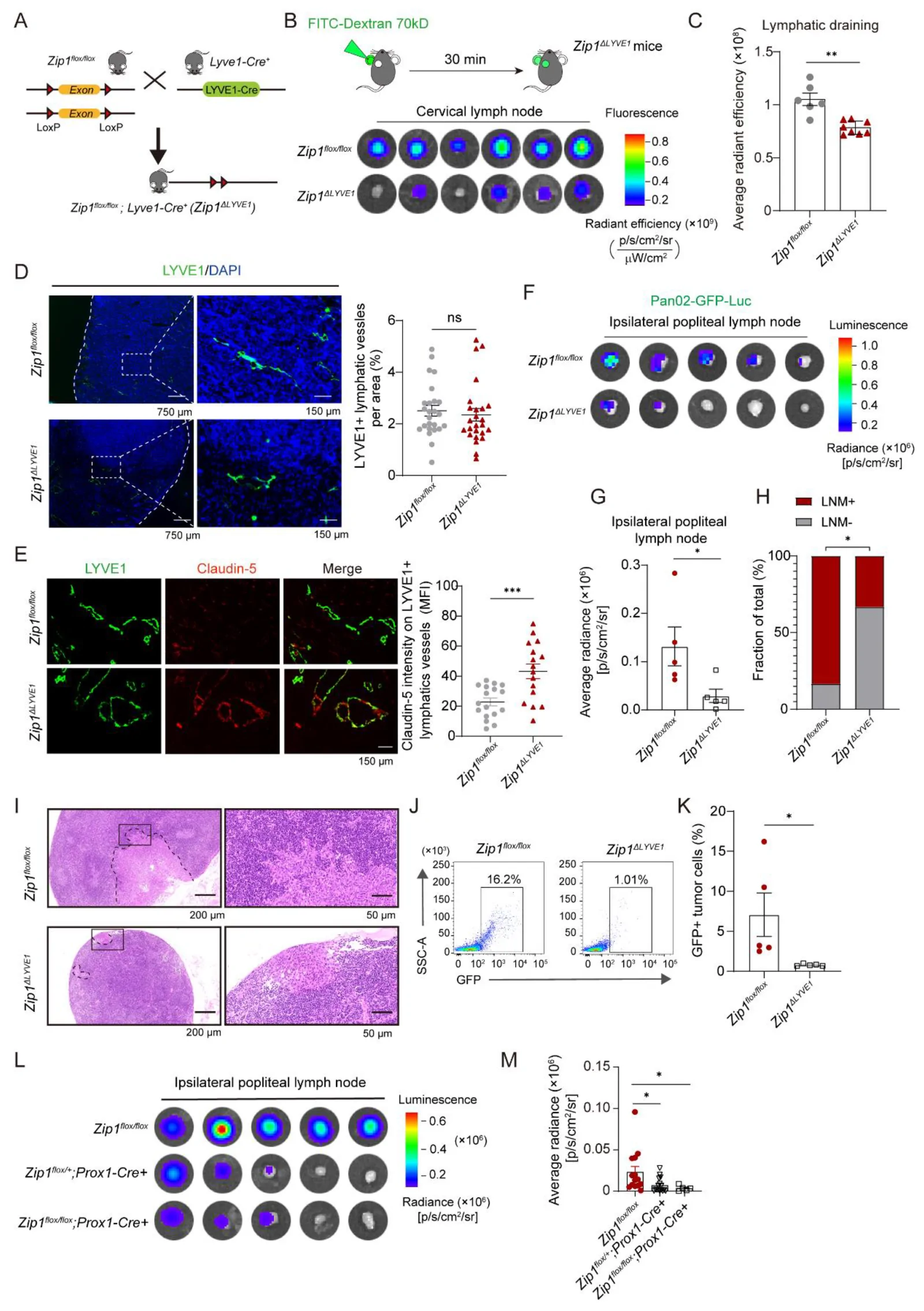
** Reduced opening of lymphatic endothelial junctions and LN metastasis of pancreatic cancer in mice with lymphatic-specific deletion of ZIP1. (A)** Schematic illustration showing the generation of lymphatic-specific ZIP1 knockout mice. **(B)** Representative images showing draining of FITC-Dextran into cervical lymph nodes after injection at the ear of indicated mice. **(C)** Quantification of FITC-Dextran fluorescence in cervical lymph nodes of indicated groups (n = 6 for *Zip1^flxo/flox^*; n = 8 for *Zip1^ΔLyve1^*). **(D)** Representative images showing LYVE1^+^ lymphatic vessels in Pan02-GFP-Luc tumor sections from *Zip1^flox/flox^* and *Zip1^ΔLyve1^* mice. Scale bar, 750 μm (left) and 150 μm (right). LYVE1^+^ vessel area per field in tumors was quantified (n = 25 fields per group).** (E)** Representative images showing LYVE1 and Claudin-5 immunostaining in Pan02-GFP-Luc tumor sections from *Zip1^flox/flox^* (n = 17 fields) and *Zip1^ΔLyve1^* (n = 16 fields) mice. Scale bar, 150 μm. Claudin-5 fluorescence intensity on LYVE1^+^ lymphatics was quantified. **(F)** Metastasis in the popliteal LNs ipsilateral to the footpad tumor from *Zip1^flox/flox^* and *Zip1^ΔLyve1^* mice detected by bioluminescence. **(G)** Quantification of bioluminescence intensity of LN metastasis shown above (n = 5 mice per group).** (H)** Quantification of LNs with metastasis (LNM) in two independent animal experiments (n = 12 for each group).** (I)** Representative HE staining of the draining LN sections from Pan02-GFP-Luc tumor-bearing *Zip1^flox/flox^* and *Zip1^ΔLyve1^* mice. Scale bar, 200 μm (left) and 50 μm (right).** (J)** Flow cytometry analysis of GFP^+^ tumor cells in ipsilateral popliteal lymph nodes. **(L)** Representative images showing metastasis in the popliteal LNs ipsilateral to the footpad tumor from *Zip1^flox/flox^*, *Zip1^flox/+^;Prox1-Cre+* and *Zip1^flox/flox^; Prox1-Cre+* mice detected by bioluminescence. **(M)** Quantification of bioluminescence intensity of LN metastasis of indicated groups(n = 14 for *Zip1^flox/flox^*, n=16 for* Zip1^flox/+^;Prox1-Cre+*, n=5 for *Zip1^flox/flox^;Prox1-Cre+* mice). *p < 0.05, **p < 0.01, ***p < 0.001. See also Figure S3.

**Figure 7:**
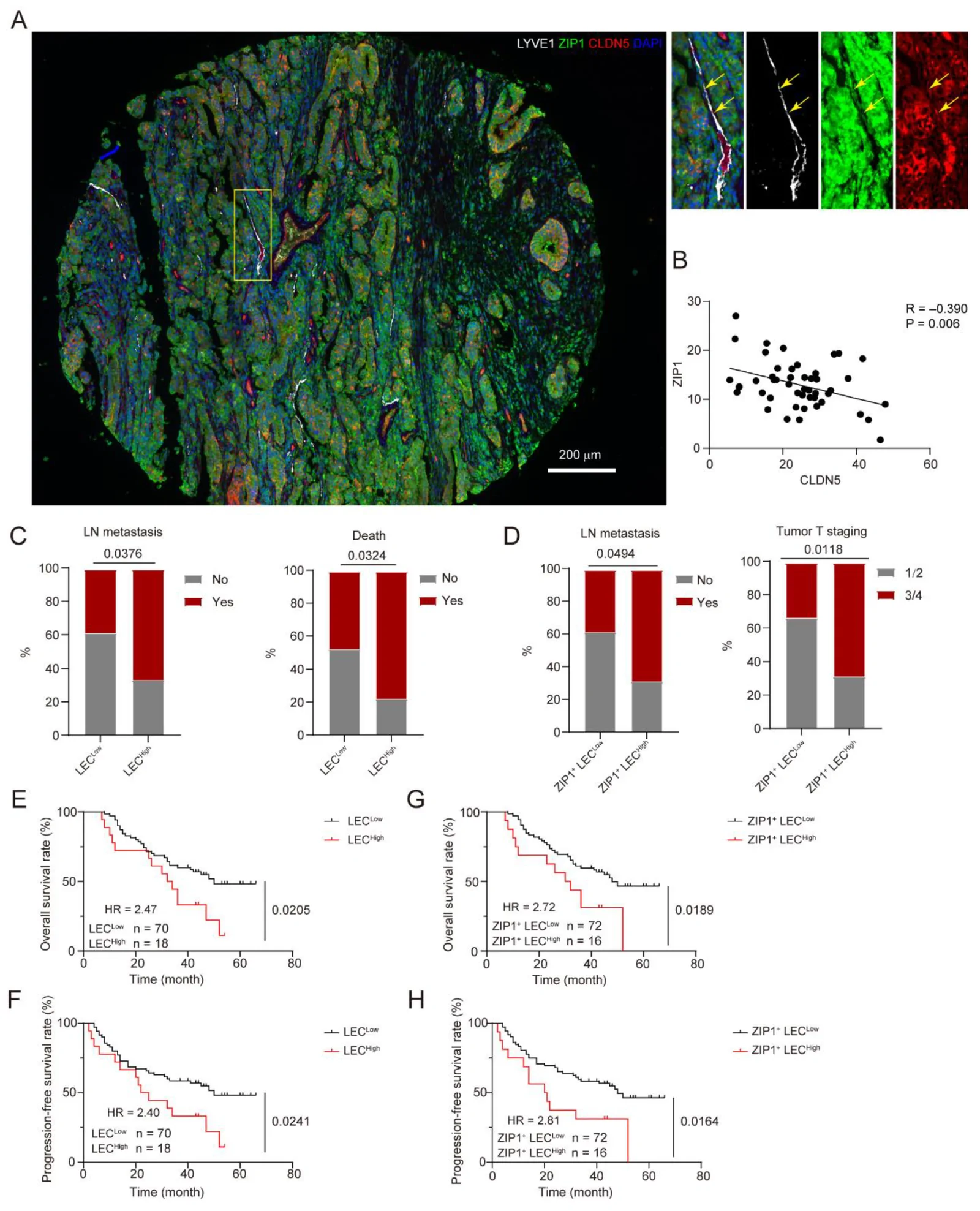
** ZIP1^+^ lymphatics were associated with LN metastasis of pancreatic cancer and poor survival in human PDAC. (A)** Representative images showing multiplex immunofluorescence staining of 88 human PDAC tissues with antibodies targeting LYVE1, ZIP1 and Claudin-5. Selected region was enlarged shown on the right. Arrows indicating the expression of ZIP1 and Claudin-5 on LYVE1^+^ lymphatics. **(B)** Pearson's correlation analysis of the expression of ZIP1 and Claudin-5 on LYVE1^+^ lymphatics. Each LYVE1^+^ lymphatics was selected and the MFI of ZIP1 and Claudin-5 was measured from 18 HPFs of two patients. A fitting line created by simple linear regression was shown. **(C)** The association of LEC with LN metastasis and the death of patients. LEC was identified with LYVE1 staining. Fisher's exact test. (**D**) The association of ZIP1^+^ LEC with LN metastasis and tumor T staging. Fisher's exact test. (**E, F**) Overall survival (**E**) and progression-free survival (**F**) of cancer patients with LEC^Low^ and LEC^High^. LEC was identified with LYVE1 staining. Log-Rank test. HR, hazard ratio. (**G, H**) Overall survival (**G**) and progression-free survival (**H**) of cancer patients with ZIP1^+^ LEC^Low^ and ZIP1^+^ LEC^High^. Log-Rank test. See also Table S1, S2.

## Data Availability

Raw data of RNA-seq have been deposited in the NGDC database (CRA015207). This paper does not report original code. Any additional information required to reanalyze the data reported in this paper is available from the corresponding author upon request.
