# Supplementary material for: ZIP1-dependent lymphatic adaptations facilitate lymph node metastasis of pancreatic cancer
Source: Int J Biol Sci. 2026 Sep 10;22(14):8119–38. doi: 10.7150/ijbs.134874 (PMC13618232; doi:10.7150/ijbs.134874)
Supplement: Supplementary file 1 [file ijbsv22p8119s1.pdf]

Supplemental information

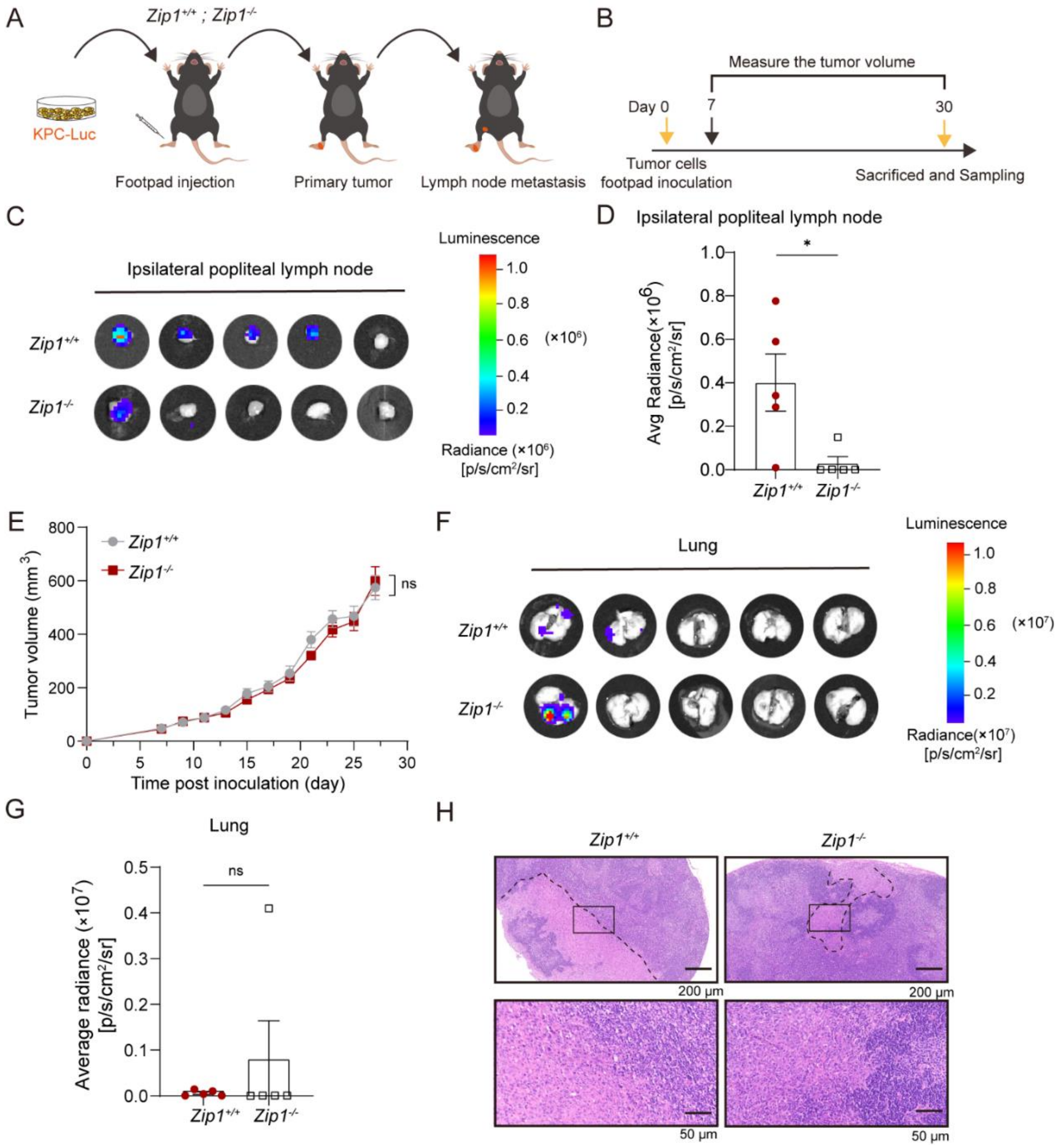

Figure S1. Host ZIP1 deficiency reduces LN metastasis of KPC-luc tumor cells. (A, B) Schematic representation of animal experiments of popliteal LN metastasis. KPC-luc cells were inoculated into the footpad of *Zip1*$^{+/+}$ and *Zip1*$^{-/-}$ mice. After 30 days, mice were sacrificed and tumor metastasis was examined. **(C)** Metastasis in the popliteal LNs ipsilateral to the footpad tumor from *Zip1*$^{+/+}$ and *Zip1*$^{-/-}$ mice detected by bioluminescence (n = 5 for each group). **(D)** Quantification of bioluminescence intensity of LN metastasis shown above. **(E)** Primary tumor growth of KPC-Luc cells monitored over time (n = 5 mice per group). **(F)** Lung metastasis *Zip1*$^{+/+}$ and *Zip1*$^{-/-}$ mice detected by bioluminescence. **(G)** Quantification of bioluminescence intensity of Lung metastasis shown above. **(H)** Representative HE staining of LN metastasis. Scale bar, 200 μm (up) and 50 μm (bottom). *$p < 0.05$. **Related to Figure 1.**

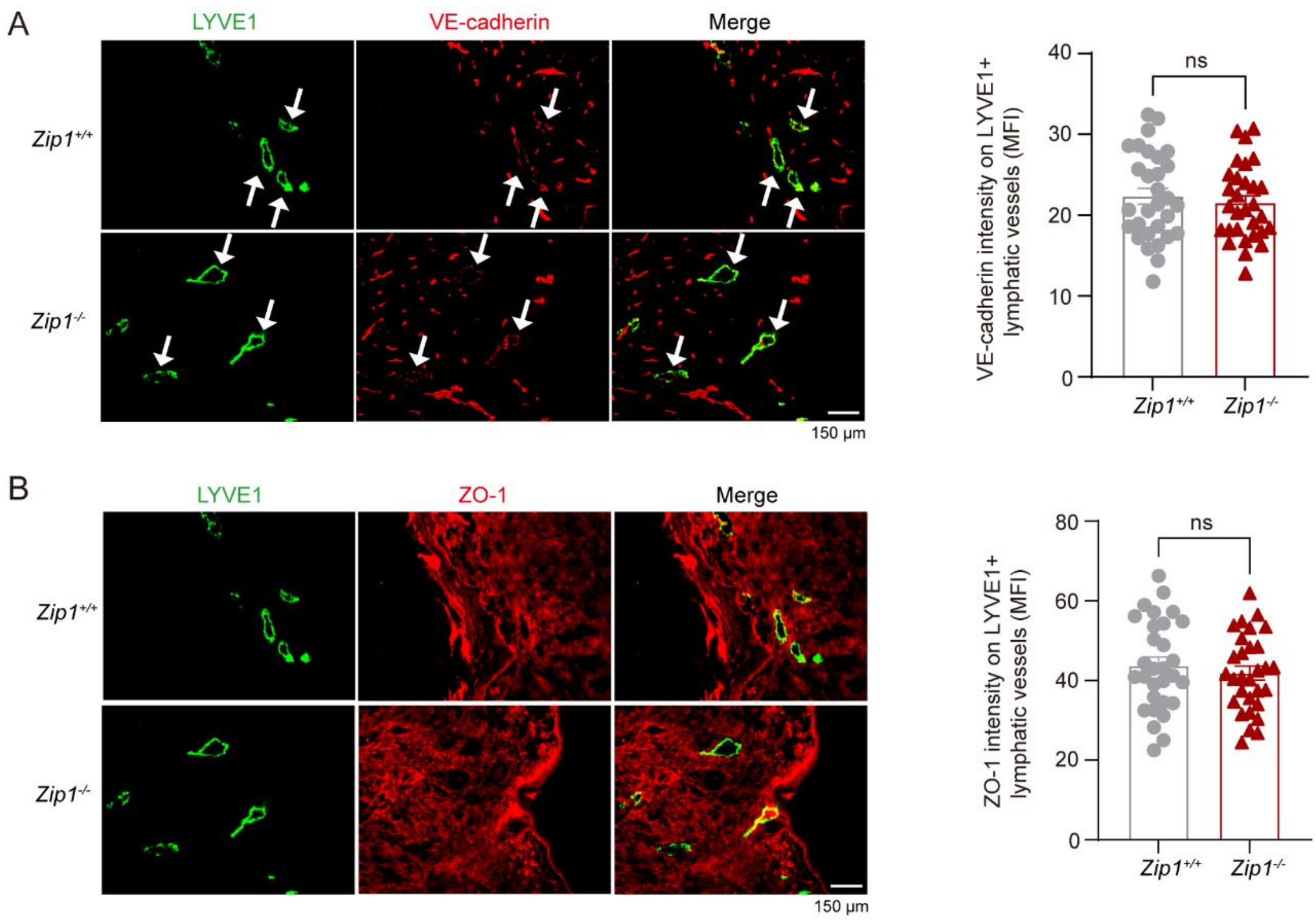

Figure S2. ZIP1 knockout does not affect the expression of VE-cadherin or ZO-1 in tumor lymphatic vessels. (A) Representative images showing LYVE1 and VE-cadherin immunostaining in Pan02-GFP-Luc tumor sections from *Zip1*$^{+/+}$ and *Zip1*$^{-/-}$ mice. Scale bar, 150 µm. VE-cadherin fluorescence intensity on LYVE1^{+} lymphatics was quantified (n = 30 fields per group). **(B)** Representative images showing LYVE1 and ZO-1 immunostaining in Pan02-GFP-Luc tumor sections from *Zip1*$^{+/+}$ and *Zip1*$^{-/-}$ mice. Scale bar, 150 µm. ZO-1 fluorescence intensity on LYVE1^{+} lymphatics was quantified (n = 30 fields per group). **Related to Figure 2.**

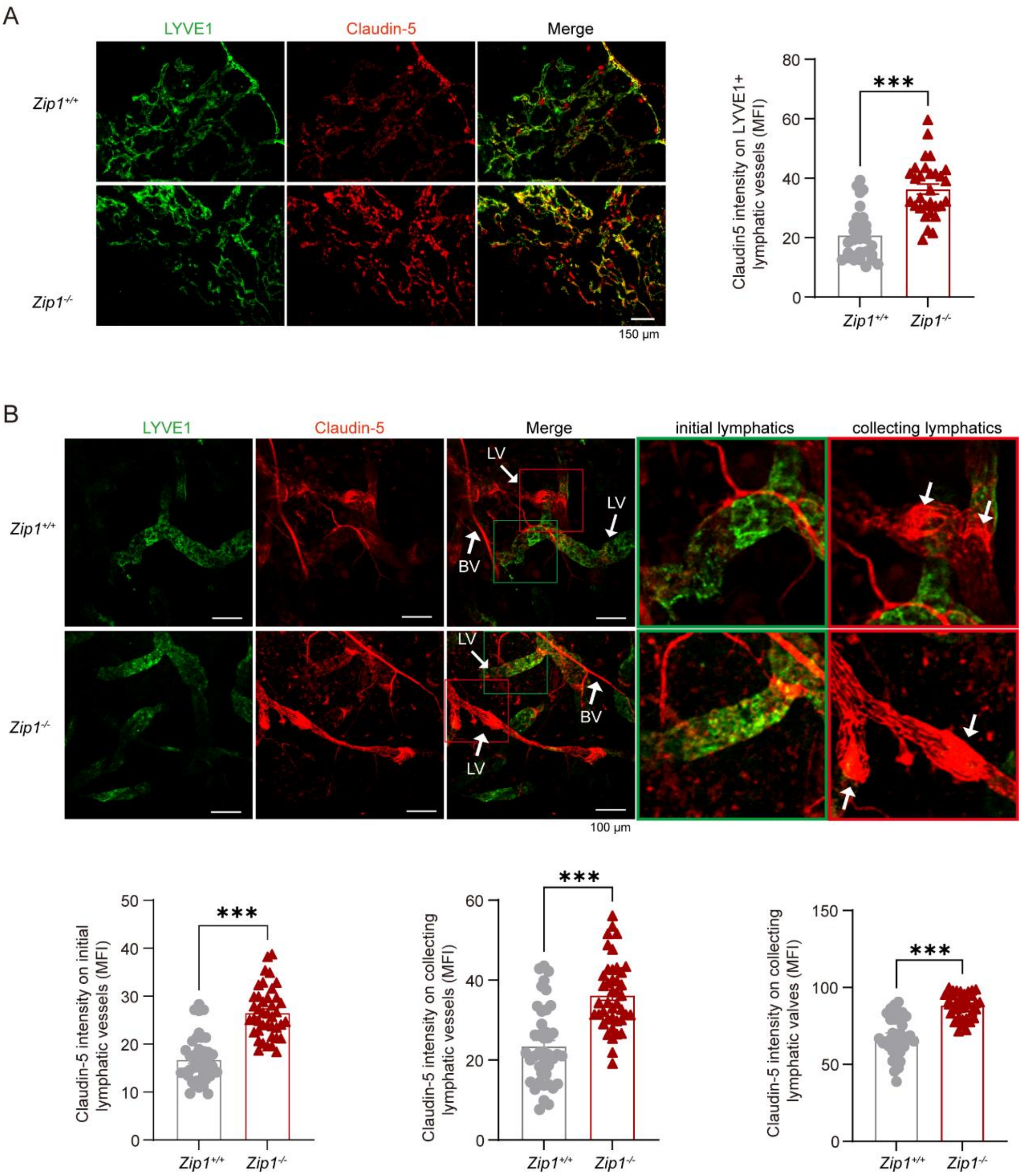

Figure S3. ZIP1 knockout significantly upregulates Claudin-5 expression in initial lymphatics, collecting vessels, and valve regions. (A) Representative images showing LYVE1 and Claudin-5 immunostaining in lymph nodes of $Zip1^{+/+}$ and $Zip1^{-/-}$ mice. Scale bar, 150 μm. Claudin-5 fluorescence intensity on $LYVE1^{+}$ lymphatics was quantified (n = 30 fields per group). **(B)** Representative images showing LYVE1 and Claudin-5 immunostaining of whole-mount mouse ear tissues from $Zip1^{+/+}$ and $Zip1^{-/-}$ mice. Scale bar, 150 μm. Claudin-5 fluorescence intensity on initial lymphatic vessels was quantified (n = 41 vessels per group). Claudin-5 fluorescence intensity on collecting lymphatic vessels was quantified (n = 42 vessels per group). Claudin-5 fluorescence intensity on collecting lymphatic valves was quantified (n = 36 fields per group). LV, lymphatic vessel. BV, blood vessel. **Related to Figure 2.**

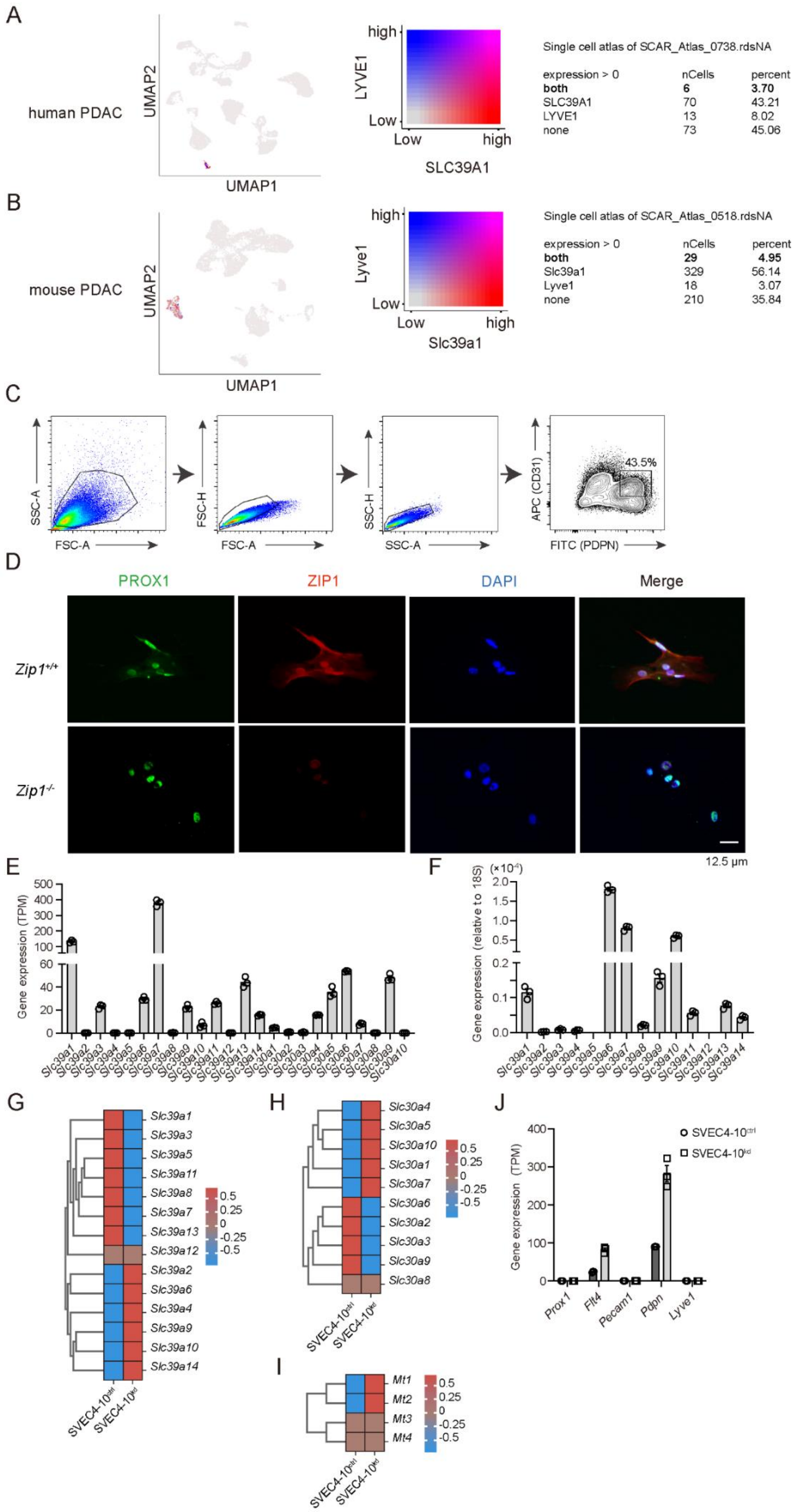
A
human PDAC
UMAP2
UMAP1
high
LYVE1
Low
Low
high
SLC39A1
Single cell atlas of SCAR_Atlas_0738.rdsNA
expression > 0 nCells percent
both 6 3.70
SLC39A1 70 43.21
LYVE1 13 8.02
none 73 45.06
B
mouse PDAC
UMAP2
UMAP1
high
Lyve1
Low
Low
high
Slc39a1
Single cell atlas of SCAR_Atlas_0518.rdsNA
expression > 0 nCells percent
both 29 4.95
Slc39a1 329 56.14
Lyve1 18 3.07
none 210 35.84
C
SSC-A
FSC-A
FSC-H
FSC-A
SSC-H
SSC-A
APC (CD31)
FITC (PDPN)
43.5%
D
PROX1
ZIP1
DAPI
Merge
Zip1+/+
Zip1-/-
12.5 μm
E
Gene expression (TPM)
Slc39a1 Slc39a2 Slc39a3 Slc39a4 Slc39a5 Slc39a6 Slc39a7 Slc39a8 Slc39a9 Slc39a10 Slc39a11 Slc39a12 Slc39a13 Slc39a14 Slc30a1 Slc30a2 Slc30a3 Slc30a4 Slc30a5 Slc30a6 Slc30a7 Slc30a8 Slc30a9 Slc30a10
F
(×10⁻⁴)
Gene expression (relative to 18S)
Slc39a1 Slc39a2 Slc39a3 Slc39a4 Slc39a5 Slc39a6 Slc39a7 Slc39a8 Slc39a9 Slc39a10 Slc39a11 Slc39a12 Slc39a13 Slc39a14
G
Slc39a1 Slc39a3 Slc39a5 Slc39a11 Slc39a8 Slc39a7 Slc39a13 Slc39a12 Slc39a2 Slc39a6 Slc39a4 Slc39a9 Slc39a10 Slc39a14
SVEC4-10ctrl
SVEC4-10kd
H
Slc30a4 Slc30a5 Slc30a10 Slc30a1 Slc30a7 Slc30a6 Slc30a2 Slc30a3 Slc30a9 Slc30a8
SVEC4-10ctrl
SVEC4-10kd
I
Mt1 Mt2 Mt3 Mt4
SVEC4-10ctrl
SVEC4-10kd
J
SVEC4-10ctrl
SVEC4-10kd
Gene expression (TPM)
Prox1 Flt4 Pecam1 Pdpn Lyve1

Figure S4. ZIP1 is expressed by LECs. (A) The expression of *ZIP1* (encoded by *SLC39A1*) in LEC from human PDAC analyzed with available single-cell atlas of SCAR database. Endothelial cells were selected and analyzed. **(B)** The expression of *Zip1* (encoded by *Slc39a1*) in LEC from spontaneous mouse PDAC analyzed with available single-cell atlas of SCAR database. Endothelial cells were selected and analyzed. **(C)** Primary $CD31^{+}PDPN^{+}$ LEC (mLEC) cells isolated from LNs were sorted by flow cytometry. **(D)** PROX1 and ZIP1 immunostaining of mLECs from $Zip1^{+/+}$ and $Zip1^{-/-}$ mice. Scale bar, 12.5 μm. **(E)** *Zip* (*Slc39a1-14*) and *Znt* (*Slc30a1-10*) family gene expression in SVEC4-10^{ctrl} cells detected by bulk-RNAseq. **(F)** *Zip* (*Slc39a1-14*) family gene expression in SVEC4-10 cells detected by RT-PCR. **(G)** Relative expression of *Zip* (*Slc39a1-14*) family genes in SVEC4-10^{ctrl} and SVEC4-10^{kd} cells detected by bulk-RNAseq. **(H)** Relative expression of *Znt* (*Slc30a1-10*) family genes in SVEC4-10^{ctrl} and SVEC4-10^{kd} cells detected by bulk-RNAseq. **(I)** Relative expression of *Mt1-4* in SVEC4-10^{ctrl} and SVEC4-10^{kd} cells detected by bulk-RNAseq. **(J)** The gene expression of *Prox1, Flt4, Pecam1, Pdpn1 and Lyve1* in SVEC4-10^{ctrl} and SVEC4-10^{kd} cells detected by bulk-RNAseq. **Related to Figure 3.**

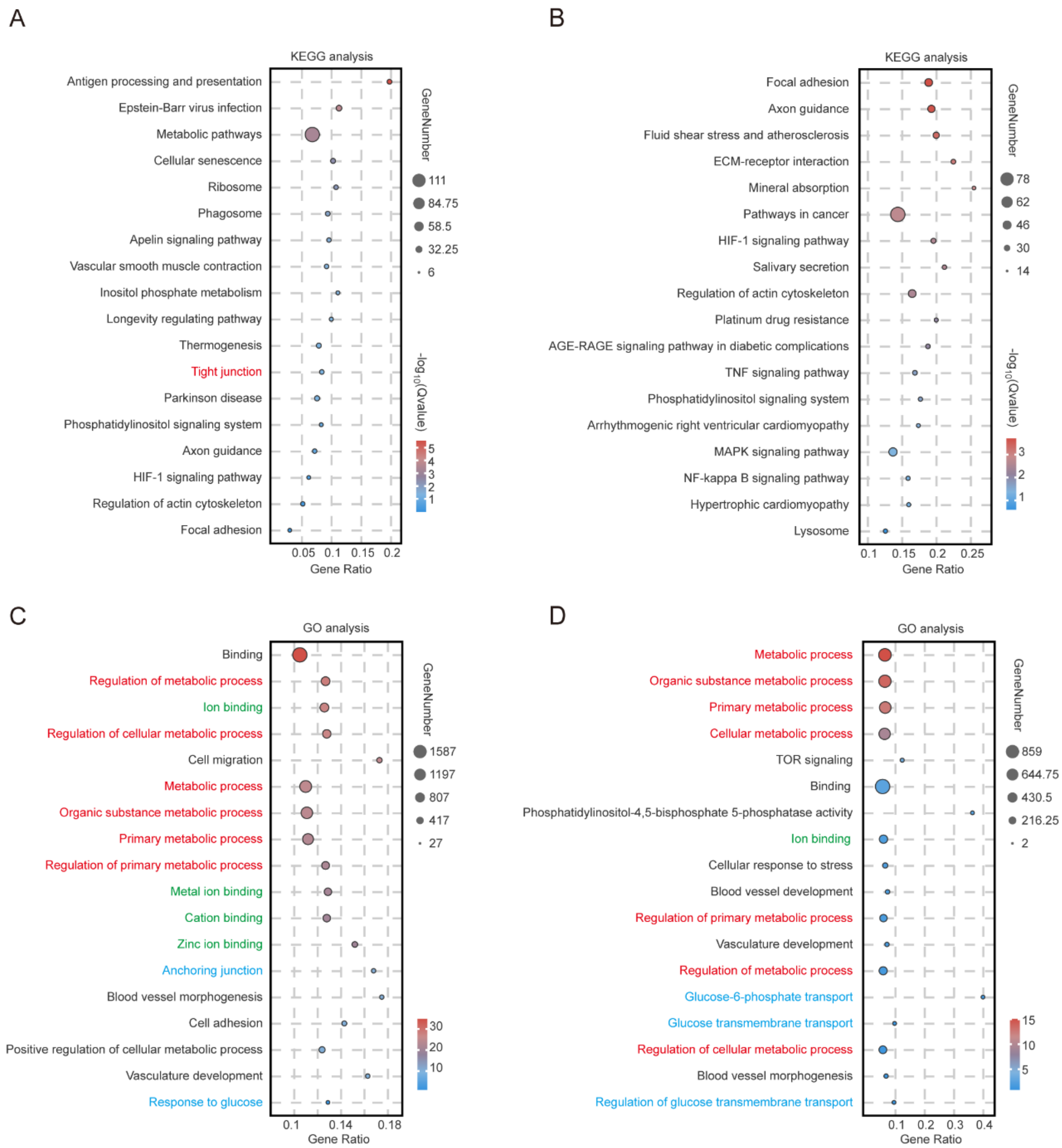

Figure S5. ZIP1 knockdown alters tight junctions and metabolic pathways in SVEC4-10 cells. (A) KEGG analysis of differential expressed genes (upregulated genes) between SVEC4-10^{ctrl} and SVEC4-10^{kd} cells showing representative enriched pathways. **(B)** KEGG analysis of differential expressed genes (downregulated genes) between SVEC4-10^{ctrl} and SVEC4-10^{kd} cells showing representative enriched pathways. **(C)** GO analysis of differential expressed genes (upregulated genes) between SVEC4-10^{ctrl} and SVEC4-10^{kd} cells showing representative enriched pathways. **(D)** GO analysis of differential expressed genes (downregulated genes) between SVEC4-10^{ctrl} and SVEC4-10^{kd} cells showing representative enriched pathways.

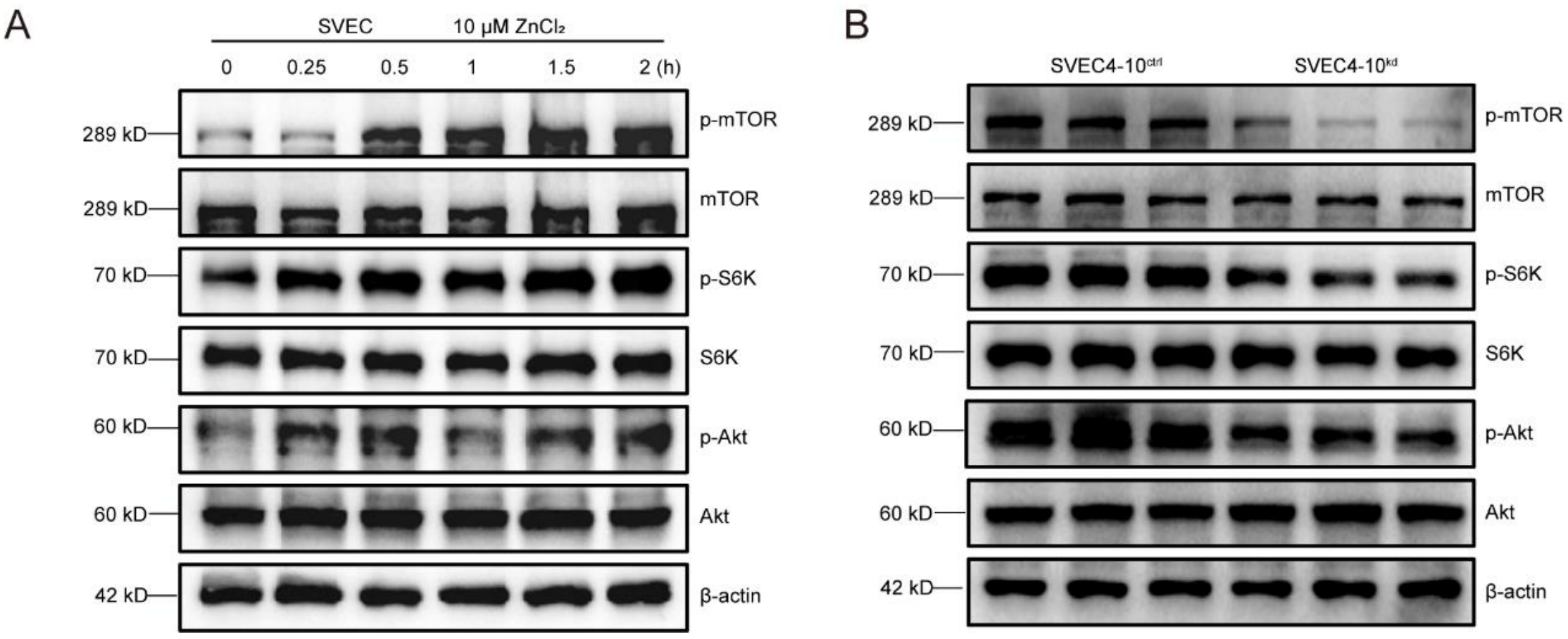

Figure S6. Zinc promotes glycolysis via activation of the Akt-mTOR-S6K signaling pathway. (A) The expression of p-mTOR, mTOR, p-S6K, S6K, p-Akt, Akt in SVEC4-10 cells stimulated with $ZnCl_2$ for the indicated durations. **(B)** The expression of p-mTOR, mTOR, p-S6K, S6K, p-Akt, Akt in SVEC4-10^{ctrl} and SVEC4-10^{kd} cells. **Related to Figure 4.**

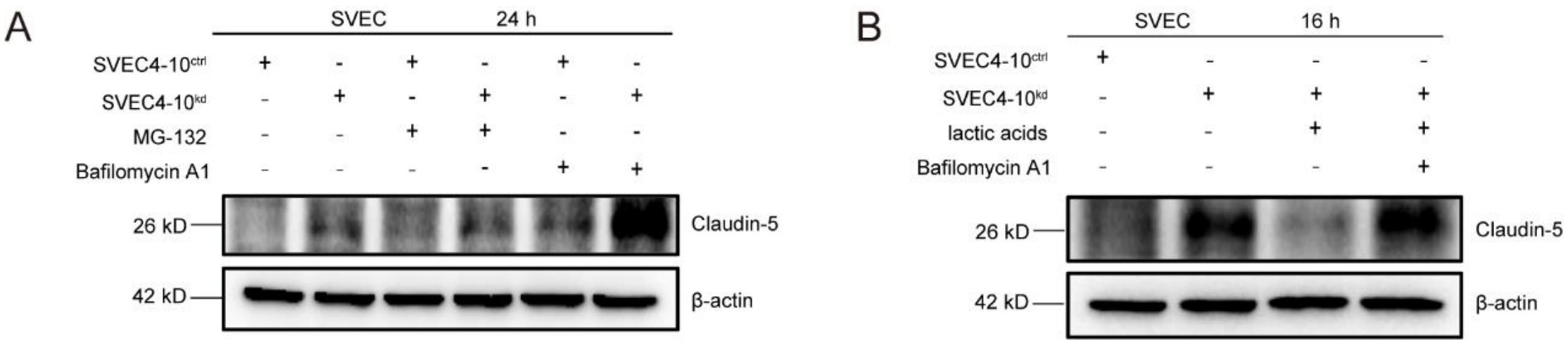

Figure S7. Claudin-5 undergoes lysosomal degradation. (A) The expression of Claudin-5 in SVEC4-10^{ctrl} and SVEC4-10^{kd} cells treated with MG-132 or Bafilomycin A1. **(B)** The expression of Claudin-5 in SVEC4-10^{ctrl} and SVEC4-10^{kd} cells treated with lactic acid or Bafilomycin A1. **Related to Figure 4.**

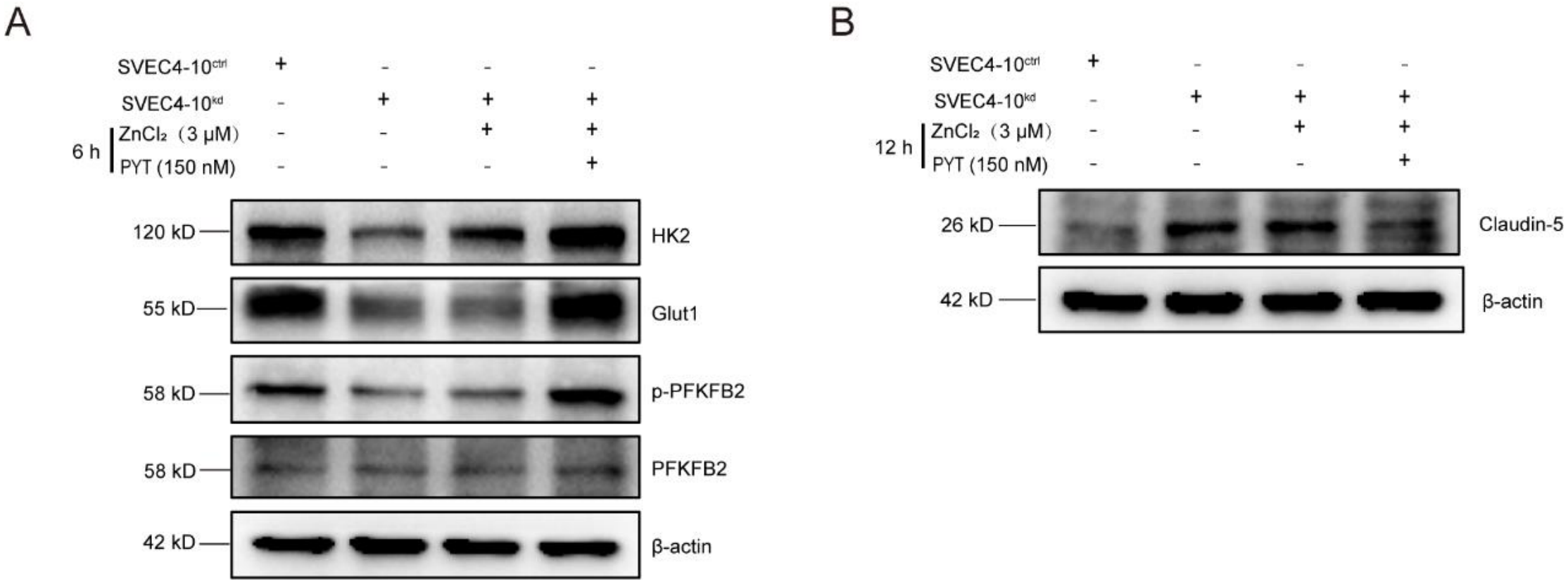

Figure S8. Zn^{2+} supplementation reverses the alterations in glycolysis and Claudin-5 expression induced by ZIP1 knockdown. (A) The expression of HK2, GLUT-1, p-PFKFB2, PFKFB2 in SVEC4-10^{ctrl} and SVEC4-10^{kd} cells treated with $ZnCl_2$ and PYT (a zinc ionophore) **(B)** The expression of Claudin-5 in SVEC4-10^{ctrl} and SVEC4-10^{kd} cells treated with $ZnCl_2$ and PYT. **Related to Figure 4.**

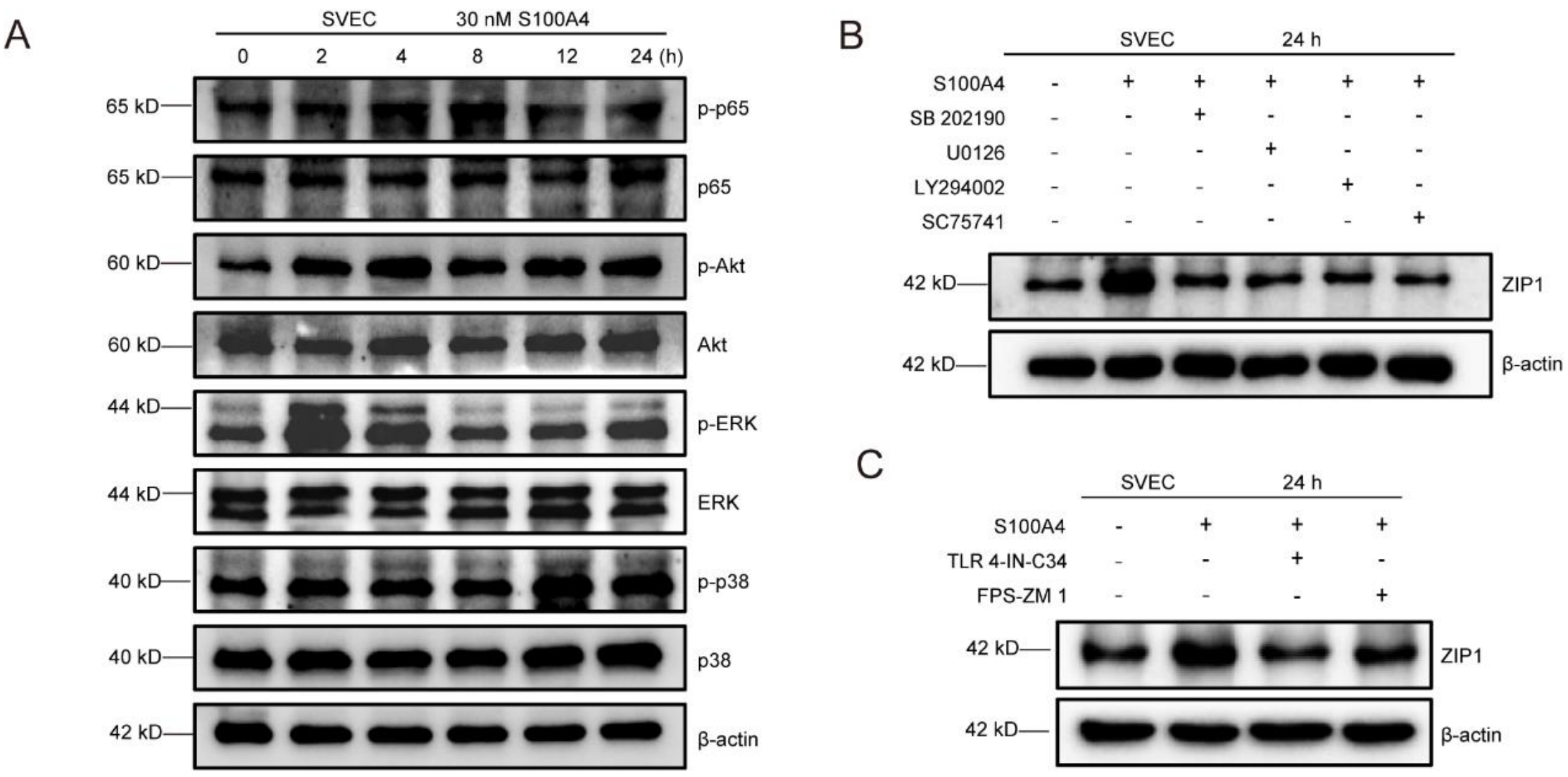

Figure S9. S100A4 upregulates ZIP1 expression via activation of the P38, ERK, P65, and AKT signaling pathways. (A) The expression of p-p65, p65, p-Akt, Akt, p-ERK, ERK, p-p38, p38 in SVEC4-10 cells stimulated with S100A4 for the indicated durations. **(B)** The expression of ZIP1 in SVEC4-10 cells treated with SB 202190 (p38 inhibitor), U0126 (ERK inhibitor), LY294002 (AKT inhibitor), or SC75741 (p65 inhibitor). **(C)** The expression of ZIP1 in SVEC4-10 cells treated with FPS-ZM1 (RAGE inhibitor) or TLR4-IN-C34 (TLR4 inhibitor). **Related to Figure 5.**

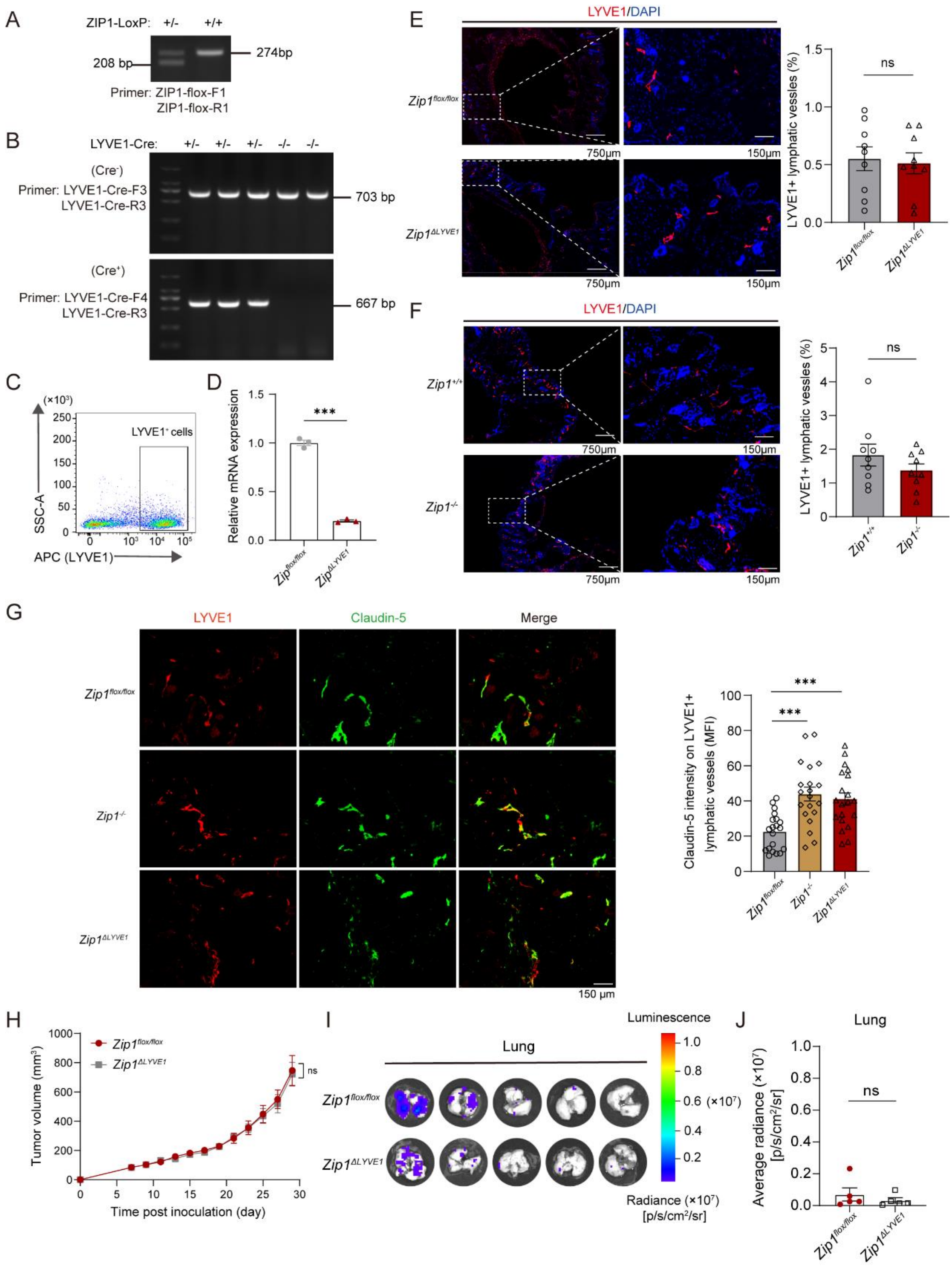

Figure S10. The expression of Claudin-5 is enhanced in lymphatic vessels from *Zip1$^{\Delta Lyve1}$* mice. (A) Genotyping of ZIP1-LoxP alleles with genomic DNA extracted from mouse tails. A pair of primers was used to detect the wild-type allele (208 bp) and the ZIP1-LoxP allele (274 bp) as indicated. **(B)** Genotyping of LYVE1-Cre alleles with genomic DNA extracted from mouse tails. Two pairs of primers were used for amplification of the wild-type allele (703 bp) and LYVE1-Cre allele (667 bp) as indicated. **(C)** Flow cytometry sorting of LYVE1^{+} cells from lymph nodes. **(D)** The mRNA expression of *Zip1* in LYVE1^{+} cells from *Zip1$^{flox/flox}$* and *Zip1$^{\Delta Lyve1}$* mice. **(E)**

Representative images showing LYVE1$^+$ lymphatic vessels in the mouse skin from *Zip1*$^{flox/flox}$ and *Zip1*$^{\Delta Lyve1}$ mice. Scale bar, 750 µm (left), 150 µm (right). LYVE1$^+$ vessel density was quantified (n = 9 fields per group). **(F)** Representative images showing LYVE1$^+$ lymphatic vessels in the mouse skin from *Zip1*$^{+/+}$ and *Zip1*$^{-/-}$ mice. Scale bar, 750 µm (left), 150 µm (right). LYVE1$^+$ vessel density was quantified (n = 9 fields per group). **(G)** Representative images showing LYVE1 and Claudin-5 immunostaining in the mouse skin from *Zip1*$^{flox/flox}$, *Zip1*$^{-/-}$ and *Zip1*$^{\Delta Lyve1}$ mice. Scale bar, 150 µm. Claudin-5 fluorescence intensity on LYVE1$^+$ lymphatics was quantified (n = 20 vessels per group). **(H)** Primary tumor growth of Pan02-GFP-Luc cells in *Zip1*$^{flox/flox}$ and *Zip1*$^{\Delta Lyve1}$ mice (n = 5 mice per group). **(I)** Lung metastasis *Zip1*$^{flox/flox}$ and *Zip1*$^{\Delta Lyve1}$ mice detected by bioluminescence. **(J)** Quantification of bioluminescence intensity of lung metastasis shown above. ***$p < 0.001$. **Related to Figure 6.**

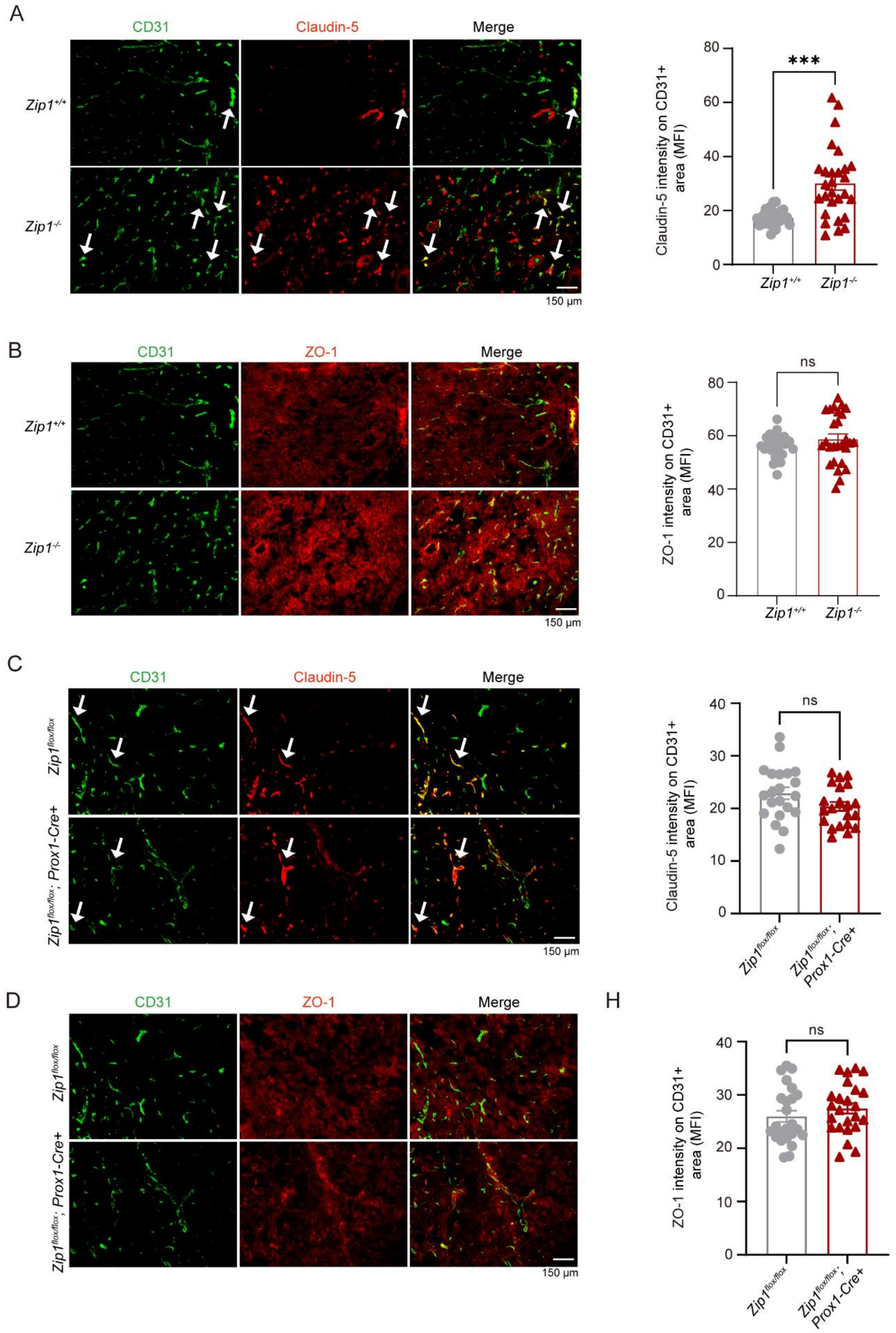

Figure S11. ZIP1 knockout upregulates Claudin-5 expression in mouse tumor vascular endothelium. (A) Representative images showing CD31 and Claudin-5 immunostaining in Pan02-GFP-Luc tumor sections from *Zip1*$^{+/+}$ and *Zip1*$^{-/-}$ mice.

Scale bar, 150 µm. Claudin-5 fluorescence intensity was quantified in $CD31^+$ areas (n = 21 fields per group). **(B)** Representative images showing CD31 and ZO-1 immunostaining in Pan02-GFP-Luc tumor sections from *Zip1*$^{+/+}$ and *Zip1*$^{-/-}$ mice. Scale bar, 150 µm. ZO-1 fluorescence intensity was quantified in $CD31^+$ areas (n = 21 fields per group). **(C)** Representative images showing CD31 and Claudin-5 immunostaining in Pan02-GFP-Luc tumor sections from *Zip1*$^{flox/flox}$ and *Zip1*$^{flox/flox}$*; Prox1-Cre+* mice Scale bar, 150 µm. Claudin-5 fluorescence intensity was quantified in $CD31^+$ areas (n = 21 fields per group). **(D)** Representative images showing CD31 and ZO-1 immunostaining in Pan02-GFP-Luc tumor sections from *Zip1*$^{flox/flox}$ and *Zip1*$^{flox/flox}$*; Prox1-Cre+* mice Scale bar, 150 µm. ZO-1 fluorescence intensity was quantified in $CD31^+$ areas (n = 21 fields per group). **Related to Figure 6.**

A

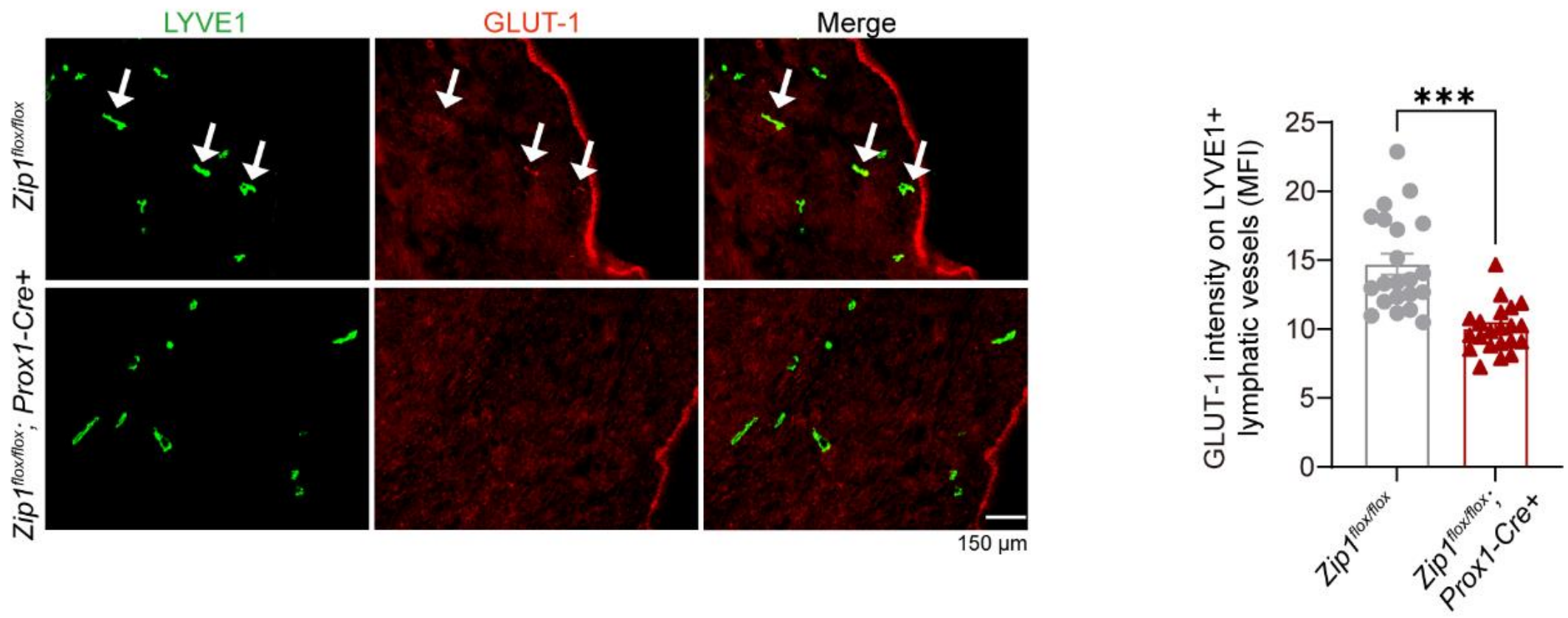

Figure S12. ZIP1 knockdown reduces GLUT-1 expression in tumor lymphatic vessels. (A) Representative images showing LYVE1 and GLUT-1 immunostaining in Pan02-GFP-Luc tumor sections from *Zip1$^{flox/flox}$* and *Zip1$^{flox/flox}$; Prox1-Cre+* mice. Scale bar, 150 μm. GLUT-1 fluorescence intensity on LYVE1^{+} lymphatics was quantified (n = 21 fields per group). **Related to Figure 6.**

Table S1. The association of LEC with clinical parameters.

		LEC^{Low}	*LEC*^{High}	*P*
Gender	F	29	4	0.1760
	M	41	14	
Age	<=60	33	9	>0.9999
	>60	37	9	
T	1/2	46	7	0.0576
	3/4	24	11	
N	N	43	6	*0.0376
	Y	27	12	
M	0	59	14	0.4975
	1	11	4	
TNM	1/2	48	11	0.5814
	3/4	22	7	
Death	N	37	4	*0.0324
	Y	33	14	
Relapse	N	44	10	0.5963
	Y	26	8	

LEC, lymphatic endothelial cell. M, male. F, female. N, no. Y, yes. *P < 0.05, Fisher's exact test. Related to Figure 7.

Table S2. The association of ZIP1^{+} LEC with clinical parameters.

		ZIP1^{+} LEC^{Low}	*ZIP1^{+} LEC^{High}*	*P*
Gender	F	29	4	0.1557
	M	43	12	
Age	<=60	34	8	>0.9999
	>60	38	8	
T	1/2	48	5	*0.0118
	3/4	24	11	
N	N	44	5	*0.0494
	Y	28	11	
M	0	61	12	0.4607
	1	11	4	
TNM	1/2	50	9	0.3806
	3/4	22	7	
Death	N	37	4	0.0945
	Y	35	12	
Relapse	N	47	7	0.1557
	Y	25	9	

LEC, lymphatic endothelial cell. M, male. F, female. N, no. Y, yes. *P < 0.05, Fisher's exact test. Related to Figure 7.

Table S3. List of primer sequences. Related to Methods.

Primer name	Forward primer (5′–3′)	Reverse primer (5′–3′)
Zip1	TGCATGTGACGGTAAGCATTG	CGCCCAGGTTATAGCTCTGTTTT
S100a4	AGCTCAAGGAGCTACTGACCAG	GCTGTCCAAGTTGCTCATCACC
Csf2	AACCTCCTGGATGACATGCCTG	AAATTGCCCCGTAGACCCTGCT
Il6	TAGTCCTTCCTACCCCAATTTCC	TTGGTCCTTAGCCACTCCTTC
Tnf	GGTGCCTATGTCTCAGCCTCTT	GCCATAGAACTGATGAGAGGGAG
Vegfc	CCTGAATCCTGGGAAATGTGCC	CGATTCGCACACGGTCTTCTGT
Claudin-5	TGACTGCCTTCCTGGACCACAA	CATACACCTTGCACTGCATGTGC
Tgfb1	TGATACGCCTGAGTGGCTGTCT	CACAAGAGCAGTGAGCGCTGAA
18s	ACCGCAGCTAGGAATAATGGA	CAAATGCTTTCGCTCTGGTC
